# NLRP4 renders pancreatic cancer resistant to olaparib through promotion of the DNA damage response and ROS-induced autophagy

**DOI:** 10.1038/s41419-024-06984-0

**Published:** 2024-08-26

**Authors:** Mingming Xiao, Jing Yang, Mingwei Dong, Xiaoqi Mao, Haoqi Pan, Yalan Lei, Xuhui Tong, Xiaoning Yu, Xianjun Yu, Si Shi

**Affiliations:** 1 Department of Pancreatic Surgery, Fudan University Shanghai Cancer Center, Shanghai, 200032 China; 2 Department of Oncology, Shanghai Medical College, Fudan University, Shanghai, 200032 China; 3 Shanghai Pancreatic Center Institute, Shanghai, 200032 China; 4 Pancreatic Center Institute, Fudan University, Shanghai, 200032 China

**Keywords:** Cancer, Cancer

## Abstract

Olaparib has been approved as a therapeutic option for metastatic pancreatic ductal adenocarcinoma patients with BRCA1/2 mutations. However, a significant majority of pancreatic cancer patients have inherent resistance or develop tolerance to olaparib. It is crucial to comprehend the molecular mechanism underlying olaparib resistance to facilitate the development of targeted therapies for pancreatic cancer. In this study, we conducted an analysis of the DepMap database to investigate gene expression variations associated with olaparib sensitivity. Our findings revealed that NLRP4 upregulation contributes to increased resistance to olaparib in pancreatic cancer cells, both in vitro and in vivo. RNA sequencing and Co-IP MS analysis revealed that NLRP4 is involved in the DNA damage response and autophagy pathway. Our findings confirmed that NLRP4 enhances the capacity for DNA repair and induces the production of significant levels of reactive oxygen species (ROS) and autophagy in response to treatment with olaparib. Specifically, NLRP4-generated mitochondrial ROS promote autophagy in pancreatic cancer cells upon exposure to olaparib. However, NLRP4-induced ROS do not affect DNA damage. The inhibition of mitochondrial ROS using MitoQ and autophagy using chloroquine (CQ) may render cells more susceptible to the effects of olaparib. Taken together, our findings highlight the significant roles played by NLRP4 in the processes of autophagy and DNA repair when pancreatic cancer cells are treated with olaparib, thereby suggesting the potential therapeutic utility of olaparib in pancreatic cancer patients with low NLRP4 expression.

## Introduction

Pancreatic cancer, a highly lethal disease originating in the exocrine pancreas, has a dismal prognosis, with a mere 10% of individuals managing to survive beyond 5 years postdiagnosis [1, 2]. The disease has often progressed to locally advanced or metastatic stages by the time it is detected, primarily due to its commonly asymptomatic presentation during the initial phases, which consequently restricts the pool of patients who can undergo surgical resection [3]. The predominant therapeutic strategy for the majority of pancreatic cancer patients involves the administration of chemotherapy [2]. Despite advancements in conventional treatment modalities such as surgery, chemotherapy, and radiation, the overall survival rate has shown minimal improvement over the past 30 years.

Pancreatic cancer is characterized by the manifestation of genomic instability, which can be ascribed to the acquisition of mutations in various DNA repair mechanisms, either through inheritance or acquired alterations [3–5]. Currently, researchers are exploring the feasibility of employing inhibitors that specifically target crucial proteins implicated in the DNA damage response (DDR) to selectively affect cancer cells [6–10]. This strategy capitalizes on the extensive mutational burden in cancer cells and the concept of synthetic lethality. For instance, poly(ADP-ribose) polymerase inhibitors (PARPi) have been approved for use in the therapeutic management of pancreatic cancers characterized by BRCA mutations [10, 11].

PARPi exhibit a range of efficacy in individuals with BRCA mutations, those with homologous recombination deficiency (HRD), and even those without HRD. Tumors lacking HR capabilities, commonly referred to as having the “BRCAness profile” [12, 13], have been shown to have relatively greater susceptibility to platinum-based chemotherapy and PARPis. PARPi exhibit limited effectiveness against HR-proficient tumors, a prevalent trait observed in a considerable proportion of pancreatic cancer cases [14]. This suboptimal efficacy can be attributed to the inadequate capacity of PARPi to induce synthetic lethality [15]. Although the therapeutic potential of PARPi extends beyond the treatment of individuals with HRD, the specific distinctions between individuals who exhibit positive responses to PARPi therapy and those who do not remain poorly understood. Currently, the identification of platinum sensitivity has been widely accepted as a dependable indicator of the suitability of the use of PARPi. The customization of precision medicine for individual patients necessitates further advancement and improvement of predictive biomarkers, which facilitate the identification of patients who would experience therapeutic advantages from PARPi.

Another issue that arises with the utilization of PARPi is the gradual emergence of resistance. Despite the demonstrated efficacy of PARPi, it is frequently observed that patients eventually acquire resistance through various mechanisms. These mechanisms include the restoration of HR functionality, secondary mutations, drug efflux, and the emergence of novel mutations in other DDR genes [14, 16–20]. Additional research is needed to investigate strategies aimed at eradicating resistance to PARPi.

This study provides evidence that NLRP4, a constituent of the nucleotide-binding and oligomerization domain-like receptor (NLR) family, imparts resistance to olaparib in pancreatic cancer cells, both in vitro and in vivo. Mechanistically, NLRP4 facilitates DNA repair and triggers elevated levels of reactive oxygen species (ROS) and autophagy, which seem to play roles in the development of resistance to PARPi.

## Methods

### Cell lines

Capan-1, BxPC-3 and HEK293T cells were acquired from the Chinese Academy of Sciences Cell Bank in Shanghai, China. HEK293T cells were grown in 10% FBS DMEM. BxPC-3 cells were maintained in RPMI-1640 medium supplemented with 10% FBS. Capan-1 cells were grown in IMDM with 10% FBS. All cells were maintained in a 37 °C incubator with 5% CO_2_.

### Plasmids and shRNA

NLRP4 plasmids were purchased from Tsingke Biotechnology Co., Ltd. The following shRNAs were used: shNLRP4-1 (5’-CCGGGCAAATGACTTTGCAGCTTGACTCGAGTCAAGCTGCAAAGTCATTTGCTTTTTT-3’) and shNLRP4-2 (5’-CCGGGCTTGGAACATAACCTTAAGACTCGAGTCTTAAGGTTATGTTCCAAGCTTTTTT-3’). shSirt7-1: 5′-GCCTGAAGGTTCTAAAGAA-3′ and shSirt7-2: 5′-TTCTTTAGAACCTTCAGGC-3′.

### Quantitative real-time PCR (qRT‒PCR)

TRIzol reagent (Thermo Fisher Scientific) was used to extract total RNA. A NanoDrop 2000 (Thermo Fisher Scientific) was used to measure the concentration and quality of the RNA. TB GreenTM Fast qPCR Mix (Takara) was used to amplify the cDNA areas of interest after the RNA was reverse-transcribed using a PrimeScript RT Kit (Takara, Japan). The 2^−ΔCt^ method was used to calculate the relative gene expression levels after normalization to GAPDH levels. The primers were as follows: GAPDH forward 5′-GAGAAGGCTGGGGCTCATTT-3′, reverse 5′-AGTGAT GGCATGGACTGTGG-3′. NLRP4 forward 5′-ACACAAAGACCTATCAAGCTCAC-3′, reverse 5′- AAAAGGCGGTCCAAATGGTCA-3′. NOXO1 forward 5′-TGGAAACCTATTCTCGGAGGC-3′, reverse 5′- GGTGCGAAGAAGCCAGTGAT-3′.

### Western blotting

Cells were lysed on ice using lysis buffer (Beyotime Biotechnology) supplemented with protease inhibitors and phosphatase inhibitors (Beyotime Biotechnology). The cell lysate was then centrifuged at 16,000 × *g* for 10 min at 4 °C, and the resulting supernatant was used in the immunoblotting analysis. Antibodies against the following proteins were used: LC3I/II (14600-1-AP, Proteintech), GAPDH (60004-1-Ig, Proteintech), p62 (207305, Abcam), H3 (1791, Abcam), NOXO1 (97788, Abcam), NLRP4 (NB100-56156, Novus), γ-H2AX (80312, Cell Signaling Technology), Sirt7 (12994-1-AP, Proteintech), and β-actin (20536-1-AP, Proteintech). The bands were visualized with a gel documentation system (Bio-Rad).

### Cell proliferation assay

The IC50 values of olaparib and the relative cell viability were determined using the MTS test. After cell adhesion, Capan-1 and BxPC-3 cells were treated with the relevant reagents in 96-well plates (2000 cells per well). Afterward, each well received 20 μl of MTS reagent, and the cells were incubated for 4 h at 37 °C. The optical absorbance at 490 nm was determined with a microplate reader.

### Colony formation

The cells were cultured for 14 days, and the colonies were fixed and stained with crystal violet stain. Colonies containing more than 50 cells were counted as surviving clones and normalized to the plating efficiency.

### Apoptosis assay

The cells were cultured in 6-well plates and exposed to olaparib for a duration of 48 h. The assessment of cellular apoptosis was conducted with flow cytometry analysis using Annexin V and 7-AAD labeling.

### RNA-seq and data analysis

RNA-seq was performed in accordance with a previous protocol [21]. Briefly, control and NLRP4-knockdown Capan-1 cells were treated with DMSO or olaparib for 48 h. The total RNA was isolated and extracted from the cells with an RNeasy Plus Mini Kit (QIAGEN). The sequencing libraries were prepared using an Illumina TruSeq Stranded Total RNA/Ribo-Zero Sample Prep Kit using high-quality total RNA with an Agilent Bioanalyzer RIN value greater than 7.0. A quantity ranging from 500 to 1000 nanograms (ng) of ribosomal RNA-depleted total RNA was fragmented by treatment with RNase III at 37 degrees Celsius for a duration of 10–18 min. Following this, RNase III was deactivated by exposing the sample to a temperature of 65 degrees Celsius for 10 min. Fragments of sizes ranging from 50 to 150 base pairs were selected with the FlashPAGE denaturing PAGE-fractionator, a product manufactured by Thermo Fisher Scientific. Following this step, the fragments were subjected to overnight ethanol precipitation. Subsequently, the RNA obtained was subjected to directed ligation, followed by reverse transcription and treatment with RNase H. Differential expression analysis were conducted using the R package ‘DESeq2’ (v1.40.2). mRNAs with |log2FC| > 1 and an adjusted *p* value < 0.05 for the test group compared to the control group were considered significant.

### Co-IP following liquid chromatography–tandem mass spectrometry analysis (Co-IP MS)

For coimmunoprecipitation (Co-IP), the cell lysate containing overexpressed Flag-NLRP4 protein was incubated with Flag antibodies and protein A/G Sepharose beads overnight at 4 °C. The proteins contained inside the beads were subjected to disruption using a solution consisting of 8 M urea in 50 mM NH_4_HCO_3_ supplemented with 1 mM NaF, 1 mM Na_3_VO_4_, and a protease inhibitor cocktail. The proteins were subjected to reduction using a 10 mM solution of dithiothreitol (DTT) for a duration of 30 min at 37 °C. Following this, alkylation was carried out using a 40 mM solution of iodoacetamide (IAA) from Sigma‒Aldrich for 45 min at room temperature in the dark. Subsequently, the removal of urea was accomplished using filter-aided sample preparation (FASP) using a 10 Kd filter obtained from Millipore. The proteins were digested by incubation with trypsin at a ratio of 1:50 (protease:protein) at 37 °C for one night. The digested peptides were quantified with the Pierce Quantitative Colorimetric Peptide Assay (Thermo Fisher Scientific). The peptides produced from each fraction were subjected to analysis using nano-LC‒MS/MS. In the context of bioinformatics analysis [22], the researchers conducted functional enrichment analysis by using PANTHER (http://www.pantherdb.org/) for protein class classification. Additionally, we used the R/bioconductor package clusterProfiler (version: 3.16.1) to perform Kyoto Encyclopedia of Genes and Genomes (KEGG) pathway enrichment. The visualization of the KEGG pathway map was performed using the Cytoscape program (version 3.8.0) in conjunction with the KEGGscape plugin (version 0.9.0).

### Silver staining

The interaction proteins were visualized using a silver staining kit according to the manufacturer’s protocol (Thermo Fisher Scientific, Waltham, MA).

### Protein-protein docking

The amino acid sequences of human Sirt7 (ID: Q9NRC8) and NLRP4 (ID: Q96MN2) were obtained from the UniProt database. Docking studies of Sirt7 and NLRP4 were conducted using HDOCK, and protein-protein interactions were visualized using PyMOL.

### Cell subcellular fractionation assay

Distinct cellular compartments were isolated using a commercial Subcellular Protein Fractionation Kit (78840, Thermo Scientific).

### Chromatin immunoprecipitation (ChIP) assay

Approximately 4 × 10^6^ pancreatic cancer cells were crosslinked using 1% formaldehyde, and the reaction was quenched with 0.125 M glycine. Subsequently, cells were pelleted, resuspended, and subjected to chromatin digestion using Micrococcal Nuclease to fragment DNA. Protein-DNA complexes were then immunoprecipitated using SimpleChIP Chromatin IP buffers according to the manufacturer’s instructions (56383, CST). The immunoprecipitation was performed using an anti-H3K18ac antibody (1191, Abcam).

### Analysis of autophagic flux

To assess autophagic flow, mRFP-GFP-LC3 plasmids were used to label and monitor LC3 proteins. The cells were subjected to transfection with mRFP-GFP-LC3 for 24 h, followed by treatment with olaparib for an additional 48 h. Fluorescence images were captured using a confocal laser scanning microscope (Leica). The yellow puncta, which are a combination of the GFP signal and RFP signal, indicated the presence of early autophagosomes. On the other hand, the red puncta, which only display the RFP signal, indicated the presence of late autolysosomes.

### Analysis of intracellular ROS levels

The intracellular levels of ROS were assessed using a 2,7-dichlorodihydrofluorescein diacetate (DCFH-DA, Beyotime) assay following the established protocol. The fluorescence signal was quantified using FASC analysis and visualized by fluorescence microscopy.

### Mitochondrial dysfunction assay

To evaluate mitochondrial dysfunction, the mitochondrial membrane potential was measured using a commercially available mitochondrial membrane potential test kit (Abcam). The quantification of the fluorescent signal was performed using FASC analysis.

### Analysis of mitochondrial ROS levels

Mitochondrial ROS levels were measured using the MitoSOX™ Red mitochondrial superoxide indicator test kit from Invitrogen. FASC analysis was used to quantify the fluorescence signal, and fluorescence microscopy was used to visualize the results.

### Transmission electron microscopy analysis

Thin slices with a thickness of 90 nm were examined with a JEOL 1200EX transmission electron microscope at an accelerating voltage of 80 kV.

### Immunofluorescence and confocal microscopy

Cells were cultivated in a 12-well plate containing glass transparencies. The cells were fixed with 4% paraformaldehyde for 30 min, permeabilized with 0.2% Triton X-100 for 5 min, blocked with blocking buffer (10% BSA in PBS) for 30 min, incubated with primary antibody for 12 h, and then incubated with secondary fluorescent antibodies for 1 h. The cell nuclei were stained for 5 min with DAPI. Protein localization was analyzed using a Leica confocal microscope.

### Animal study

Four- to six-week-old, 16- to 18-gram BALB/c nude mice were obtained from Fudan University and maintained in the SPF Animal Centre. All animal care and experimental procedures were conducted in accordance with the National Institutes of Health Guide for the Care and Use of Laboratory Animals and were approved by Fudan University’s Animal Care and Ethics Committee. Olaparib (50 mg/kg) was administered daily for in vivo drug trials. Each tumor was measured every 3 days.

### Immunohistochemical (IHC) staining

Tissues were fixed in 4% formaldehyde at room temperature overnight and then embedded in paraffin. Embedded 4-mm paraffin sections were deparaffinized with xylene. Using EDTA- or citrate-based antigen retrieval, the GTVisionTM III detection system (GeneTech) was used to conduct IHC staining according to established protocols. Images were taken with an Olympus BX61 microscope. The expression of the stained markers was assessed using a histologic score (H score).

### Statistical analysis

SPSS was used to conduct Student’s *t* test or one-way ANOVA to compare two or multiple groups, respectively. Data are presented as the means and standard deviations, and differences with *p* value (*p < 0.05, **p < 0.01, ***p < 0.001) are regarded as significant.

## Results

### NLRP4 expression promotes the proliferation and olaparib resistance of pancreatic cancer

**Fig. 1:**
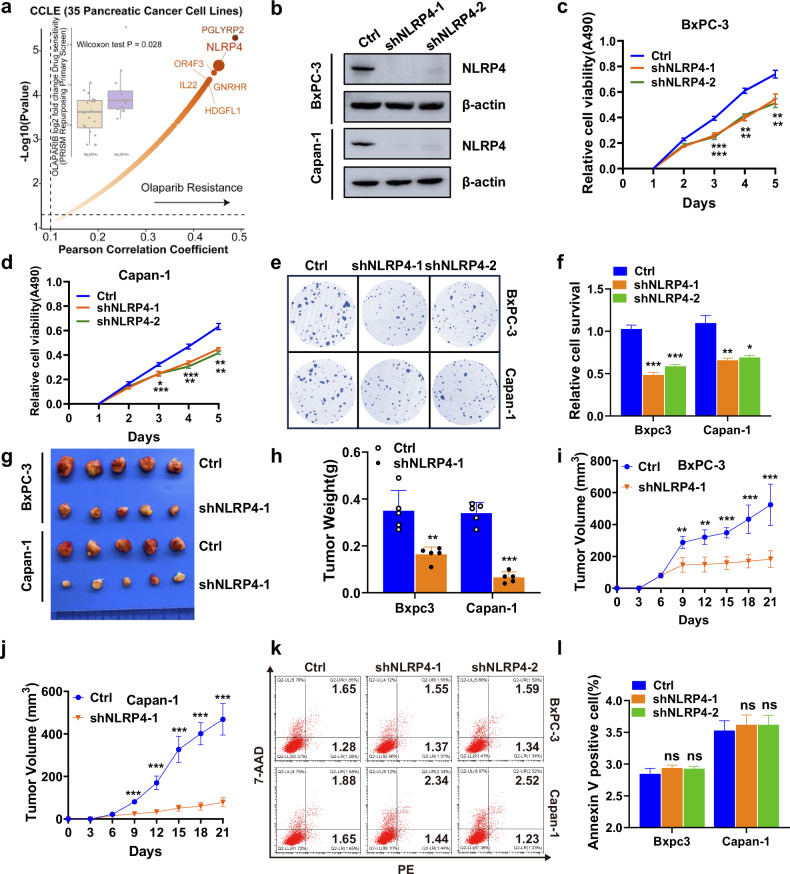
NLRP4 expression promotes pancreatic cancer cell proliferation. **a** Volcano plot showing the suppression of NLRP4 led to a greater susceptibility to olaparib in pancreatic cancer cell lines. **b** Immunoblot of NLRP4 in control and NLRP4-knockdown BxPC-3 and Capan-1 cells. BxPC-3 and Capan-1 cells were stably transfected with shNC, shNLRP4-1 and shNLRP4-2, and then MTS assays (**c**, **d**) and colony formation assays (**e**, **f**) were performed. ***p < 0.001. **p < 0.01. The analysis of significant differences was performed with Student’s *t* test. **g**–**j** Control and NLRP4-knockdown BxPC-3 and Capan-1 cells were injected into the left flank of nude mice. Tumor volumes were measured every 3 days. Tumors were harvested on day 21, photographed and weighed. Tumor weights are shown in (**h**), BxPC-3 tumor volumes are shown in (**i**), and Capan-1 tumor volumes are shown in (**j**). Data are shown as the mean ± SD (*n* = 5). ***p < 0.001. The analysis of significant differences was performed with Student’s *t* test. ***p < 0.001. **p < 0.01. **k**, **l** Flow cytometry analysis of annexin V/7-ADD staining in the indicated cell lines. Data are presented as the mean ± SD from three independent experiments. The analysis of significant differences was performed with Student’s *t* test.

To facilitate a comprehensive comparison of differences in olaparib sensitivity, an analysis of the DepMap database (https://depmap.org/portal/download/) was conducted. The results of this investigation show that the suppression of NLRP4 led to a greater susceptibility to olaparib in pancreatic cancer cell lines (Fig. 1a). To explore the role of NLRP4 in pancreatic cancer cell lines, the expression of NLRP4 was silenced in BxPC-3 (BRCA wild type) and Capan-1 (possessing BRCA2 mutation) cells (Fig. 1b and Extended Data Fig. 1a). The findings of the MTS and colony formation assays revealed that the depletion of NLRP4 suppressed the in vitro proliferation of pancreatic cancer cells, as depicted in Fig. 1c–f. Additionally, the downregulation of NLRP4 expression hindered the growth of pancreatic cancer tumors in vivo (Fig. 1g–j). It is important to emphasize that NLRP4 did not exert any discernible influence on cellular apoptosis, as evidenced in Fig. 1k, l.

**Fig. 2:**
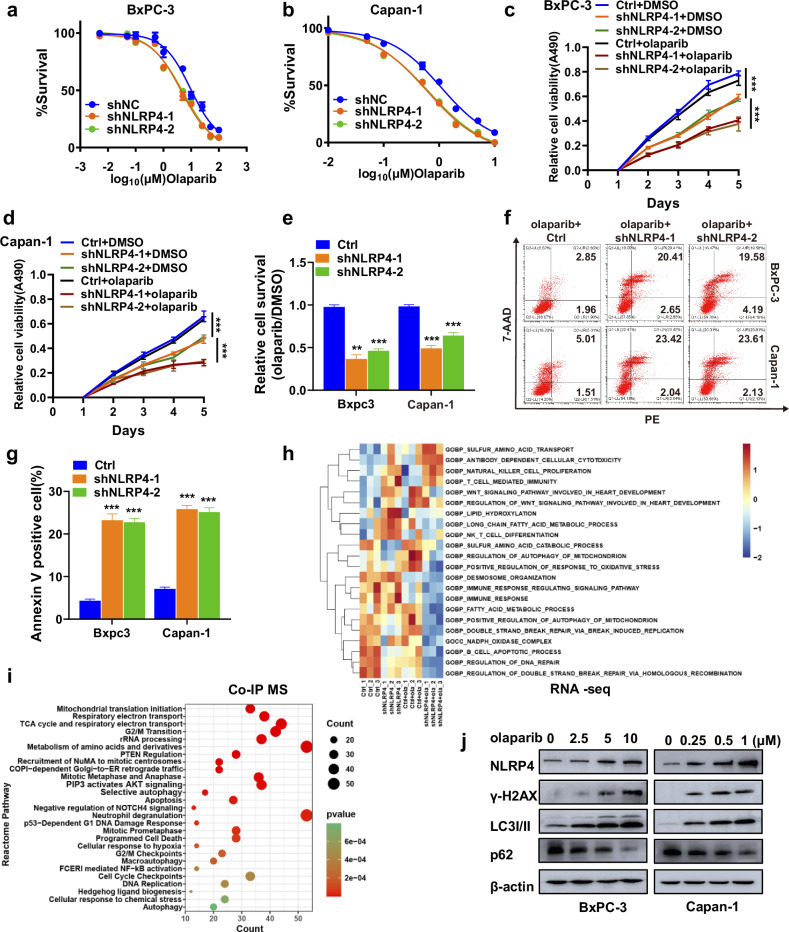
NLRP4 expression induced olaparib resistance in pancreatic cancer cells. **a**, **b** MTS assays were performed to calculate the IC50 of olaparib in the indicated cells. **c**, **d** The indicated cells were treated with DMSO or olaparib (5 μM olaparib for BxPC-3 cells and 500 nM olaparib for Capan-1 cells). MTS assays were performed to investigate the effect of NLRP4 on olaparib resistance. The analysis of significant differences was performed with one-way ANOVA. ***p < 0.001. **e** The indicated cells were treated with DMSO or olaparib (5 μM olaparib for BxPC-3 cells and 500 nM olaparib for Capan-1 cells). Colony formation assays were performed to investigate the effect of NLRP4 on olaparib resistance. The analysis of significant differences was performed with Student’s *t* test. ***p < 0.001. **p < 0.01. **f**, **g** Flow cytometry results for annexin V/7-ADD staining in the indicated cell lines after exposure to DMSO or olaparib (5 μM olaparib for BxPC-3 cells and 500 nM olaparib for Capan-1 cells). The analysis of significant differences was performed with Student’s *t* test. ***p < 0.001. **h** Control or NLRP4-knockdown Capan-1 cells were treated with DMSO or olaparib (500 nM) for RNA sequencing and were subjected to GO pathway enrichment analysis. **i** Co-IP experiments were conducted in NLRP4 OE cells, followed by LC‒MS/MS analysis. **j** Control or NLRP4-knockdown BxPC-3 and Capan-1 cells were treated with the indicated dose of olaparib for 48 h. Western blotting was performed with the indicated antibodies.

To assess the potential impact of NLRP4 on the resistance of pancreatic cancer cells to olaparib, we administered varying doses of olaparib to NLRP4-knockdown or control stable BxPC-3 (BRCA wild type) and Capan-1 (possessing BRCA2 mutation) cell lines. The findings depicted in Fig. 2a, b and Extended Data Fig. 1b, c indicated that the downregulation of NLRP4 in both BRCA-wild-type and BRCA-mutant pancreatic cancer cells heightened their susceptibility to olaparib. Additionally, NLRP4 knockdown in pancreatic cancer cells led to a decrease in the half-maximal inhibitory concentration (IC50) values for olaparib. Moreover, the results obtained from MTS and colony formation assays demonstrated that the knockdown of NLRP4 significantly enhanced the inhibitory effect of olaparib on the proliferation of pancreatic cancer cells (Fig. 2c–e and Extended Data Fig. 1e). Additionally, an Annexin-V/7-AAD experiment was conducted, providing compelling evidence that the combination of NLRP4 knockdown and olaparib therapy led to a substantial increase in the rate of apoptosis (Fig. 2f, g). These results provide evidence that NLRP4 fosters cell proliferation and confers resistance to olaparib in both BRCA-wild-type and BRCA-mutant pancreatic cancer cells.

To investigate which pathways NLRP4 affects to induce olaparib resistance, we conducted an RNA sequencing analysis in Capan-1 cells with or without NLRP4 knocked down that were treated with DMSO or olaparib. GO and KEGG enrichment analysis showed that the DNA repair pathway and autophagy pathway were suppressed after NLRP4 knockdown treated with olaparib (Fig. 2h and Extended Data Fig. 2a, b). Additionally, the NLRP4 reactome pathway was implicated in the DNA damage response and autophagy pathway, as revealed by NLRP4 coimmunoprecipitation coupled with mass spectrometry (Co-IP MS) analysis (Fig. 2i and Extended Data Fig. 2c, d). Moreover, our investigation demonstrated that olaparib treatment resulted in the upregulation of NLRP4 expression and phosphorylation of the H2AX histone (γ-H2AX, a recognized marker of DNA damage), as well as the conversion of LC3-I to LC3-II (a well-established marker of autophagy) (Fig. 2j). These findings suggest that NLRP4 may play a role in regulating the DNA repair pathway and autophagy pathway.

### NLRP4 promotes the efficiency of DNA repair in response to olaparib

**Fig. 3:**
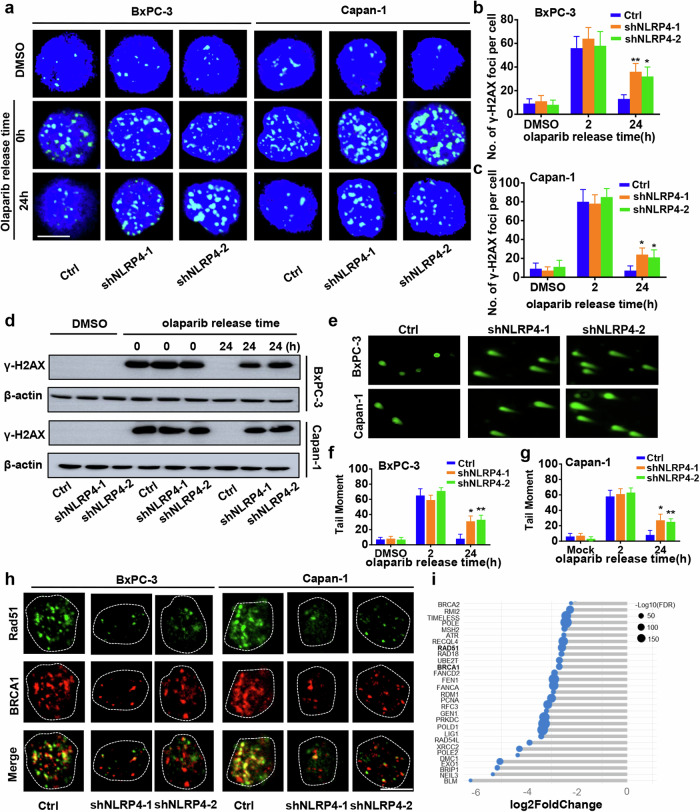
NLRP4 promotes the efficiency of DNA repair in response to olaparib exposure. **a**–**c** Representative immunofluorescence micrographs and quantification of γ-H2AX foci in the indicated cells with or without olaparib treatment (5 μM olaparib for BxPC-3 cells and 500 nM olaparib for Capan-1 cells). The analysis of significant differences was performed with Student’s *t* test. **p < 0.01, *p < 0.05. Scale bar, 5 μm. **d** Control or NLRP4-knockdown BxPC-3 and Capan-1 cells were treated with DMSO or olaparib (5 μM olaparib for BxPC-3 cells and 500 nM olaparib for Capan-1 cells) for 48 h. Western blotting was performed with the indicated antibodies. **e**–**g** Representative comet assay micrographs and quantification of tail moments in the indicated cells with or without olaparib treatment (5 μM olaparib for BxPC-3 cells and 500 nM olaparib for Capan-1 cells). The analysis of significant differences was performed with Student’s *t* test. **p < 0.01, *p < 0.05. **h** Representative immunofluorescence micrographs showing BRCA1 and Rad51 IRIF in the indicated cell lines at 6 h after olaparib treatment (5 μM olaparib for BxPC-3 cells and 500 nM olaparib for Capan-1 cells). Scale bar, 5 μm. **i** Changes in DNA repair genes in control and NLRP4-knockdown Capan-1 cells after olaparib treatment (500 nM olaparib for Capan-1 cells).

To investigate the potential influence of NLRP4 on the DNA damage response, γ-H2AX foci were examined in pancreatic cancer cells with NLRP4 knockdown and in control cells treated with either DMSO or olaparib (Fig. 3a–c). Immediately following the removal of olaparib, both control and NLRP4-knockdown cells exhibited a higher number of γ-H2AX foci. Notably, NLRP4-knockdown BxPC-3 and Capan-1 cells displayed the prominent presence of γ-H2AX foci within their nuclei 24 h after the removal of olaparib. Conversely, control cells did not have any γ-H2AX foci 24 h after the removal of olaparib. Notably, no disparity in γ-H2AX foci between the control and NLRP4-knockdown groups was observed when the cells were subjected to DMSO treatment, suggesting that NLRP4 does not influence DNA damage induction but rather impacts DNA damage repair. This finding is supported by the results obtained from the immunofluorescence assay and further bolstered by the findings from the immunoblot analysis, as shown in Fig. 3d. Additionally, a comet assay was performed to assess the extent of DNA damage in the pancreatic cancer cells. Notably, the comet tail distance of NLRP4-knockdown pancreatic cancer cells was significantly greater than that of the control group following a 24-h period of olaparib treatment (Fig. 3e, f). Intriguingly, the immunofluorescence assay revealed a reduction in the foci of BRCA1 and Rad51, two crucial proteins involved in DNA repair, in NLRP4-knockdown pancreatic cancer cells 6 h after olaparib removal (Fig. 3h and Extended Data Fig. 3a–d). Furthermore, an examination of the RNA sequencing data revealed a noteworthy decrease in the expression of genes implicated in DNA repair, including BRCA1, Rad51, within the NLRP4-knockdown cells subjected to olaparib treatment (Fig. 3i). These findings provide evidence that the NLRP4 protein is essential for DNA repair in pancreatic cancer cells exposed to olaparib.

### Pancreatic cancer cells with high NLRP4 expression exhibit enhanced levels of autophagy in response to treatment with olaparib

**Fig. 4:**
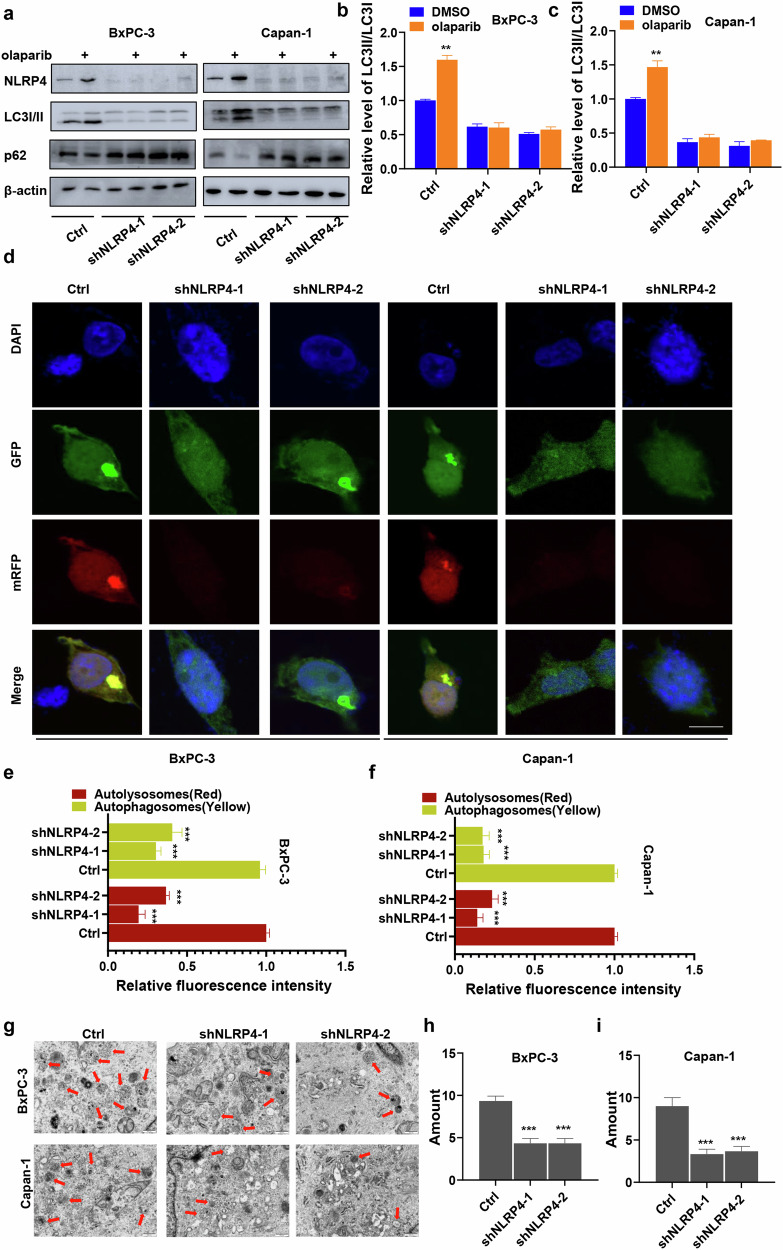
Pancreatic cancer cells with high NLRP4 expression exhibit enhanced levels of autophagy in response to olaparib exposure. **a**–**c** Control or NLRP4-knockdown BxPC-3 and Capan-1 cells were treated with DMSO or olaparib (5 μM olaparib for BxPC-3 cells and 500 nM olaparib for Capan-1 cells) for 48 h. Western blotting analysis and quantification were performed. The analysis of significant differences was performed with Student's *t* test.**p < 0.01. **d**–**f** Effect of NLRP4 on the levels of autophagic flux. The mRFP-GFP-LC3 plasmid was transfected into the indicated cells for 24 h, and then the cells were treated with olaparib (5 μM olaparib for BxPC-3 cells and 500 nM olaparib for Capan-1 cells) for 48 h. Representative images were obtained by laser scanning confocal microscopy. The average fluorescence intensity of autophagic lysosomes (yellow dots in the merged images) and autophagic lysosomes (red in the merged images) in individual cells was quantified. Scale bar, 5 μm. The analysis of significant differences was performed with Student's *t* test. ***p < 0.001. **g**–**i** TEM-based ultrastructure analysis (autophagosomes) in the indicated cells. The analysis of significant differences was performed with Student’s *t* test. ***p < 0.001.

RNA sequencing data and Co-IP MS data showed that the induction of olaparib resistance by NLRP4 involved the autophagy pathway, as seen in Fig. 2h, i. Consequently, we proceeded to evaluate the levels of the autophagy marker proteins LC3I/II and p62 to determine the potential contribution of autophagy to NLRP4-mediated resistance to olaparib. Western blot analysis showed that the inhibition of NLRP4 resulted in the suppression of the conversion of LC3-I to LC3-II conversion in BxPC-3 (1.61-fold decrease for shNLRP4-1 and 1.96-fold decrease for shNLRP4-2) and Capan-1 (2.70-fold decrease for shNLRP4-1 and 3.23-fold decrease for shNLRP4-2) cells, as shown in Fig. 4a–c, when compared to the control group.

The utilization of mRFP-GFP-LC3 in dual fluorescence enabled the visualization of autophagic flow under fluorescence microscopy [23]. Upon treatment with olaparib, the red and yellow fluorescence intensity (representing autophagic lysosomes and autophagosomes, respectively) in BxPC-3 and Capan-1 cells was reduced in NLRP4-knockdown cells compared to the levels in the control cells (Fig. 4d–f). Furthermore, a comparison of cellular ultrastructural morphology between control and NLRP4-knockdown cells was conducted using a transmission electron microscope. The NLRP4-knockdown cells exhibited reduced autophagosome formation following olaparib treatment (Fig. 4g–i). Together, our research suggests that the upregulation of autophagy in pancreatic cancer cells plays a role in the development of resistance to olaparib, which is mediated by NLRP4.

### NLRP4 induces mitochondrial ROS generation in pancreatic cancer cells in response to treatment with olaparib

**Fig. 5:**
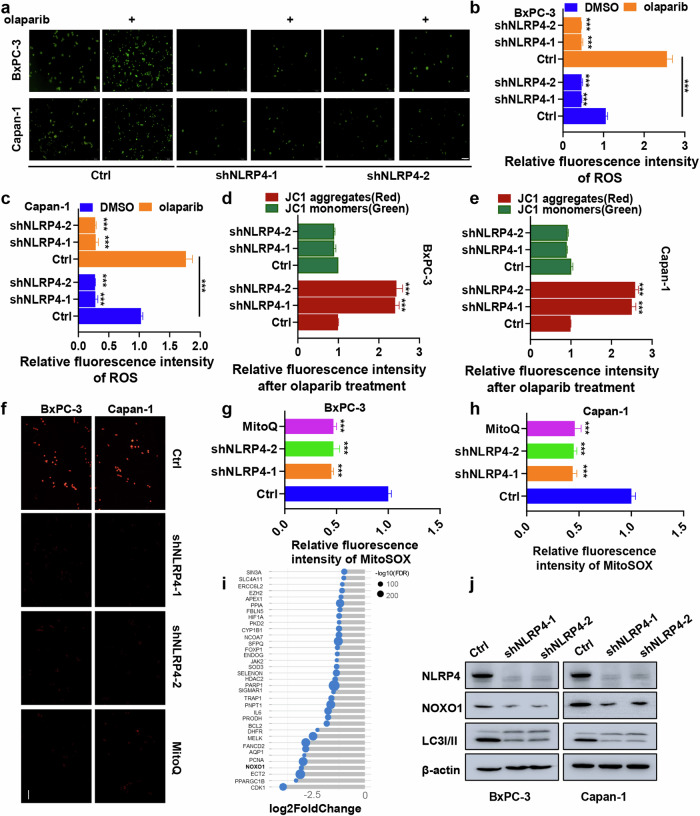
NLRP4 induces mitochondrial ROS generation in pancreatic cancer cells in response to olaparib exposure. **a** Cells treated with DMSO or olaparib (5 μM olaparib for BxPC-3 cells and 500 nM olaparib for Capan-1 cells) for 48 h were incubated with an ROS indicator. Representative images were obtained. **b**, **c** Cells treated with DMSO or olaparib (5 μM olaparib for BxPC-3 cells and 500 nM olaparib for Capan-1 cells) for 48 h were incubated with an ROS indicator, and fluorescence intensity was assessed with flow cytometry. The analysis of significant differences was performed with Student’s *t* test. ***p < 0.001. **d**, **e** Cells treated with DMSO or olaparib (5 μM olaparib for BxPC-3 cells and 500 nM olaparib for Capan-1 cells) for 48 h were incubated with JC-1 working solution, and fluorescence intensity was assessed with flow cytometry. The analysis of significant differences was performed with Student’s *t* test. ***p < 0.001. **f**–**h** Cells treated with olaparib (5 μM olaparib for BxPC-3 cells and 500 nM olaparib for Capan-1 cells) plus DMSO or MitoQ for 48 h were incubated with MitoSOX. Representative images were obtained, and the fluorescence intensity was assessed with flow cytometry. The analysis of significant differences was performed with Student’s *t* test. ***p < 0.001. **i** Changes in ROS-related genes in control and NLRP4-knockdown Capan-1 cells after olaparib treatment (500 nM olaparib for Capan-1 cells). Scale bar, 5 μm. **j** Western blotting analysis was performed for the indicated antibodies.

The results of RNA sequencing indicated a decrease in the activation of the ROS pathway in olaparib-treated NLRP4-knockdown cells (Fig. 2h). Meanwhile, the involvement of NLRP4 in the ROS pathway was demonstrated through Co-IP MS data (Fig. 2i and Extended Data Fig. 4a). Consequently, we proceeded to investigate the intracellular ROS levels in NLRP4-knockdown BxPC-3 and Capan-1 cells, both with and without olaparib treatment. Upon administration of olaparib, fluorescence microscopy and flow cytometry analysis revealed an increase in intracellular ROS levels in control BxPC-3 and Capan-1 cells (Fig. 5a–c and Extended Data Fig. 4b). However, NLRP4-knockdown cells did not exhibit an induction of ROS in response to exposure to olaparib.

The Co-IP MS data revealed a significant association between NLRP4 and various mitochondrial-related functions, including the respiratory chain complex, the oxidoreductase complex, and NADH dehydrogenase activity (Extended Data Fig. 5a). Previous studies have indicated a strong link between increased intracellular ROS levels and compromised mitochondrial function [24–26]. Thus, our objective was to examine the potential association between mitochondrial dysfunction and NLRP4-induced ROS production in the context of the response to exposure to olaparib. In NLRP4-knockdown cell lines subjected to olaparib treatment, the assessment of mitochondrial dysfunction revealed a significant decrease in the red fluorescence of JC-1 aggregates (Fig. 5d, e and Extended Data Fig. 5b), suggesting that NLRP4 induces mitochondrial impairment in response to exposure to olaparib. It is plausible that the transportation of ROS within mitochondria constitutes a positive-feedback mechanism that promotes intracellular ROS generation, potentially leading to observable mitochondrial damage. The impact of NLRP4 on the generation of ROS in the mitochondria in response to exposure to olaparib was investigated using MitoSOX staining (Fig. 5f–h and Extended Data Fig. 5c). The results showed that NLRP4-knockdown cell lines exhibited a significant decrease in mitochondrial ROS generation upon exposure to olaparib.

### NLRP4 promotes NOXO1 expression by inhibiting Sirt7 nuclear entry

To investigate NLRP4’s potential role in mitochondrial ROS induction, we performed comparative gene expression analysis in control cells treated with olaparib and NLRP4-knockdown cells treated with olaparib (Fig. 5i). This analysis revealed a significant reduction in NOXO1, a subunit of NADPH oxidase known to enhance intracellular ROS production via mitochondrial ROS generation, in NLRP4-knockdown cells. This observation was further confirmed through western blot analysis and qRT-PCR (Fig. 5j and Extended Data Fig. 6a).

**Fig. 6:**
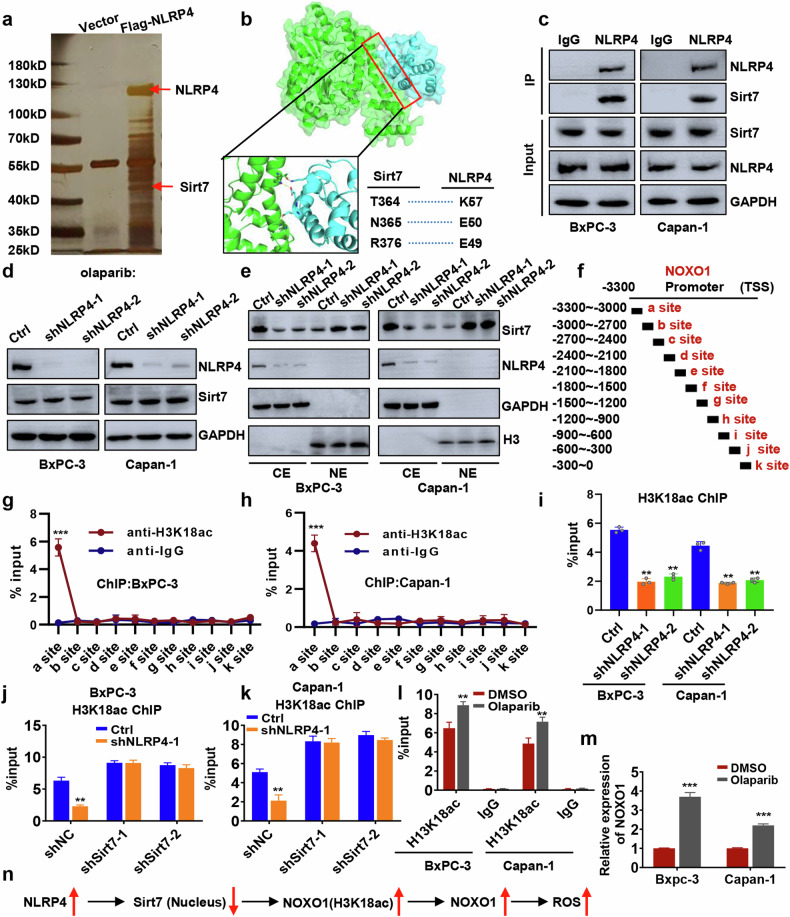
NLRP4 promotes NOXO1 expression by inhibiting Sirt7 entry into the nucleus. **a** Silver staining of NLRP4-interacting proteins. **b** Molecular dynamics simulation-derived model structure of NLRP4 bound to Sirt7. **c** Cell lysates were immunoprecipitated with the indicated antibodies. **d**, **e** Western blot analysis was performed using the indicated antibodies. **f**–**l** ChIP-qPCR assay to assess H3K18ac status at the NOXO1 genomic region. The analysis of significant differences was performed with Student's *t* test. **p < 0.01, ***p < 0.001. **m** qPCR analysis of NOXO1 expression. **n** Proposed model depicting the role of NLRP4-induced ROS.

Since NLRP4 is cytoplasmic and unable to directly perform transcriptional regulatory functions in the nucleus, we hypothesized that it modulates nuclear entry of transcription factors/repressors. We investigated NLRP4’s interactome using Co-IP MS and found that Sirt7, a transcriptional repressor, interacts with NLRP4. NLRP4 did not affect Sirt7 expression levels (Fig. 6a–d and Extended Data Fig. 6b), However, nuclear-cytoplasmic fractionation experiments indicated that NLRP4 inhibits Sirt7 nuclear localization (Fig. 6e). Given Sirt7’s role in histone H3 lysine 18 (H3K18) deacetylation, which typically reduces gene expression, we examined H3K18 acetylation (H3K18ac) levels near the NOXO1 promoter. The database revealed an enrichment of H3K18ac signals approximately 3000 bp upstream of the NOXO1 promoter region in some cell lines (Extended Data Fig. 6c). As a result, we performed the experiment on pancreatic cancer cell lines. ChIP-qPCR experiments in pancreatic cancer cell lines confirmed significant H3K18ac enrichment in NOXO1 promoter region (Fig. 6f–h and Extended Data Fig. 6d), which was diminished upon NLRP4 knockdown (Fig. 6i), suggesting NLRP4’s regulation of H3K18ac at the NOXO1 promoter. However, after inhibiting Sirt7, knocking down NLRP4 had little effect on the enrichment of the H3K18 acetylation signal in the NOXO1 promoter (Fig. 6j, k), indicating that NLRP4 modulates H3K18 acetylation of NOXO1 promoter via Sirt7. As olaparib enhances NLRP4 expression, we investigated the effect of olaparib on H3K18 acetylation in the NOXO1 promoter. We discovered that olaparib raised H3K18ac enrichment in the NOXO1 promoter and improved NOXO1 transcription (Fig. 6l, m). Overall, our findings demonstrate that NLRP4 impedes Sirt7 nuclear entry, leading to enhanced H3K18 acetylation of NOXO1 promoter and consequently promoting NOXO1 transcription (Fig. 6n).

**Fig. 7:**
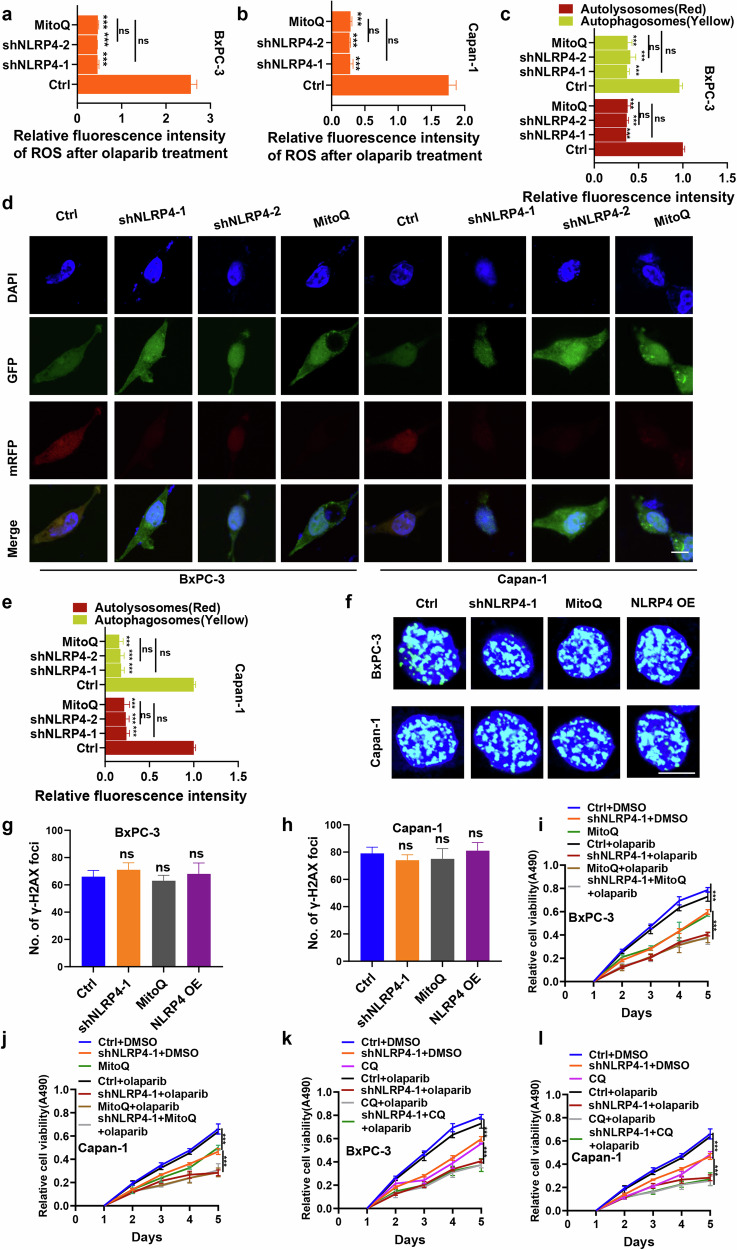
NLRP4-induced autophagy is associated with mitochondrial ROS in pancreatic cancer cells in response to olaparib exposure. **a**, **b** Cells treated with olaparib (5 μM olaparib for BxPC-3 cells and 500 nM olaparib for Capan-1 cells) plus DMSO or MitoQ for 48 h were incubated with an ROS indicator, and fluorescence intensity was assessed with flow cytometry. The analysis of significant differences was performed with one-way ANOVA. ***p < 0.001. **c**–**e** The mRFP-GFP-LC3 plasmid was transfected into the indicated cells for 24 h, and then the cells were treated with olaparib (5 μM olaparib for BxPC-3 cells and 500 nM olaparib for Capan-1 cells) plus DMSO or MitoQ for 48 h. Representative images were obtained by laser scanning confocal microscopy. The average fluorescence intensity of autophagic lysosomes (yellow dots in the merged images) and autophagic lysosomes (red in the merged images) in individual cells was quantified. The analysis of significant differences was performed with Student’s *t* test. ***p < 0.001. Scale bar, 5 μm. **f**–**h** Representative immunofluorescence micrographs and quantification of γ-H2AX foci in the indicated cells treated with olaparib (5 μM olaparib for BxPC-3 cells and 500 nM olaparib for Capan-1 cells) plus MitoQ or DMSO for 48 h. The analysis of significant differences was performed by Student’s *t* test. Scale bar 5 μm. **i**, **j** The indicated cells were treated with DMSO or olaparib (5 μM olaparib for BxPC-3 cells and 500 nM olaparib for Capan-1 cells) with or without MitoQ. MTS assays were performed. The analysis of significant differences was performed with one-way ANOVA. ***p < 0.001. **k**, **l** The indicated cells were treated with DMSO or olaparib (5 μM olaparib for BxPC-3 cells and 500 nM olaparib for Capan-1 cells) with or without CQ. MTS assays were performed. The analysis of significant differences was performed with one-way ANOVA. ***p < 0.001.

Additionally, the use of mitoquinone mesylate (MitoQ), a targeted inhibitor of mitochondrial ROS, may potentially inhibit the NLRP4-induced generation of ROS in the mitochondria. In addition, an investigation was conducted to assess the intracellular levels of ROS in NLRP4-knockdown cells that were subjected to olaparib treatment, and the inhibitory effect was compared to that of MitoQ. The findings revealed no discernible disparity in intracellular ROS levels between NLRP4-knockdown pancreatic cancer cells and control cells treated with MitoQ (Fig. 7a, b and Extended Data Fig. 7a, b). This suggests that NLRP4-induced mitochondrial ROS amplify the release of intracellular ROS in response to exposure to olaparib.

### NLRP4-induced autophagy is associated with mitochondrial ROS in pancreatic cancer cells in response to treatment with olaparib

It has been documented that autophagy is triggered by ROS [24, 27–29]. Therefore, our objective was to investigate the potential correlation between NLRP4-induced ROS and NLRP4-induced autophagy. After olaparib administration, the red and yellow fluorescence intensities (representing autophagic lysosomes and autophagosomes, respectively) were reduced in NLRP4-knockdown BxPC-3 and Capan-1 cells. However, there was no discernible difference in autophagy levels between pancreatic cancer cells treated with MitoQ and those with NLRP4 knockdown (Fig. 7c–e), suggesting that NLRP4-induced autophagy is mediated by ROS production. We subsequently investigated whether NLRP4-induced mitochondrial ROS could potentially increase DNA damage in cells treated with olaparib, as previous studies have indicated that ROS may contribute to DNA damage. To assess this, we examined the formation of γ-H2AX foci in response to olaparib in various cell groups, including control cells, NLRP4-knockdown cells, NLRP4-overexpressing pancreatic cancer cells, and MitoQ-treated cells (Fig. 7f–h). Interestingly, the levels of γ-H2AX did not significantly vary among these cell groups, suggesting that NLRP4 and mitochondrial ROS could not lead to DNA damage. Given that NLRP4 has been shown to stimulate autophagy and ROS production in pancreatic cancer cells, we conducted further investigations to explore the potential implications of these findings. The knockdown of NLRP4, the administration of MitoQ, and the use of the autophagy inhibitor CQ all contributed to the enhanced inhibitory effect of olaparib on the proliferation of pancreatic cancer cells (Fig. 7i–l). Furthermore, an Annexin-V/7-AAD experiment showed that there was a significant increase in the rate of apoptosis when NLRP4 knockdown, MitoQ, or CQ was combined with olaparib therapy (Extended Data Fig. 7c, d), suggesting that MitoQ and CQ effectively improved the NLRP4-mediated resistance of pancreatic cancer cells to olaparib.

### Pancreatic tumors with higher NLRP4 expression are resistant to treatment with olaparib in vivo

**Fig. 8:**
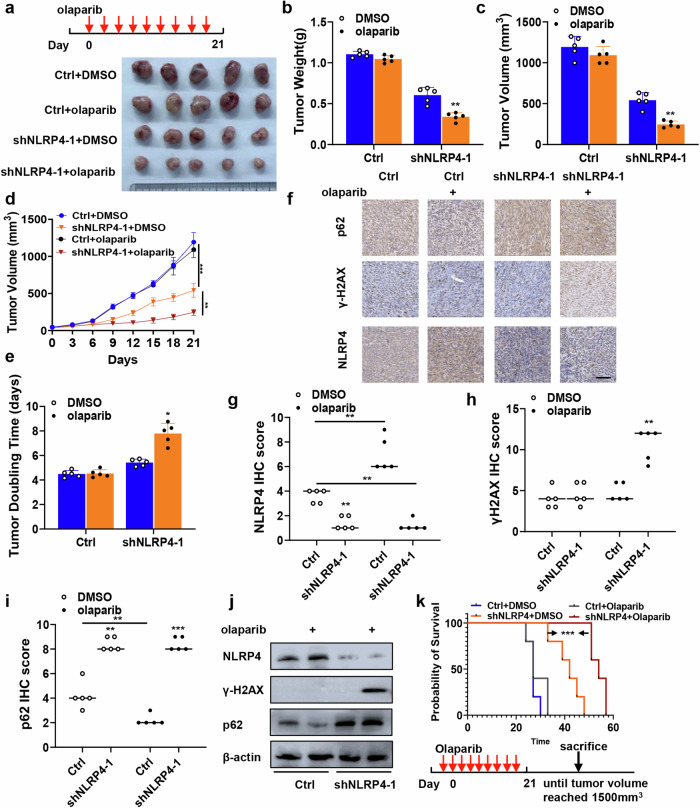
Pancreatic tumors with higher NLRP4 expression are resistant to olaparib in vivo. **a** BxPC-3 xenografts obtained from mice in different groups treated with DMSO or olaparib (50 mg/kg per day). **b** Quantification of the weight of the tumors in different groups treated with DMSO or olaparib (50 mg/kg per day). The analysis of significant differences was performed with Student’s *t* test. **p < 0.01. **c** Quantification of tumor volume on the last day in different groups treated with DMSO or olaparib (50 mg/kg per day). The analysis of significant differences was performed with Student’s *t* test. *p* < 0.05, ** *p* <0.01. **d** Growth curves of cells treated with DMSO or olaparib. The analysis of significant differences was performed with one-way ANOVA. **p < 0.01, ***p < 0.001. **e** Tumor doubling time in different groups treated with DMSO or olaparib (50 mg/kg per day). The analysis of significant differences was performed with Student’s *t* test. *p<0.05. **f**–**h** Tumors were subjected to immunological staining to detect the indicated marker. **f** Representative IHC micrographs. Scale bar, 100 μm. **g**–**i** The histological score (H score) of the indicated markers was quantified. The analysis of significant differences was performed with Student’s *t* test. *p < 0.05,**p < 0.01,***p < 0.001. **j** Western blotting analysis was performed for the indicated antibodies. **k** Kaplan–Meier survival curves for the specified groups. The *p* value was calculated by the log rank test. ***p < 0.001.

To assess the effects of NLRP4 on tumor growth, olaparib sensitivity, DNA damage response, and autophagy in vivo, we conducted experiments using nude mice that were implanted with pancreatic cancer xenografts. Treatment with olaparib significantly suppressed the growth of NLRP4-knockdown BxPC-3 and Capan-1 tumors (Fig. 8a–e and Extended Data Fig. 8a–e). Furthermore, we assessed the impact of NLRP4 on the expression of tumor-associated proteins in xenografts using immunohistochemistry (Fig. 8f–i and Extended Data Fig. 8f–i) and western blot analysis (Fig. 8j). Consistent with our cell-based findings, olaparib induces upregulation of NLRP4. NLRP4 knockdown following olaparib therapy resulted in elevated levels of γ-H2AX. Olaparib inhibits p62 expression, which is elevated following NLRP4 knockdown. Moreover, olaparib treatment significantly extended the survival of NLRP4 knockdown mice (Fig. 8k and Extended Data Fig. 8j).

### Complementation of NLRP4 rescues DNA repair defects and autophagy levels in NLRP4-knockdown pancreatic cancer cells and results in olaparib resistance

**Fig. 9:**
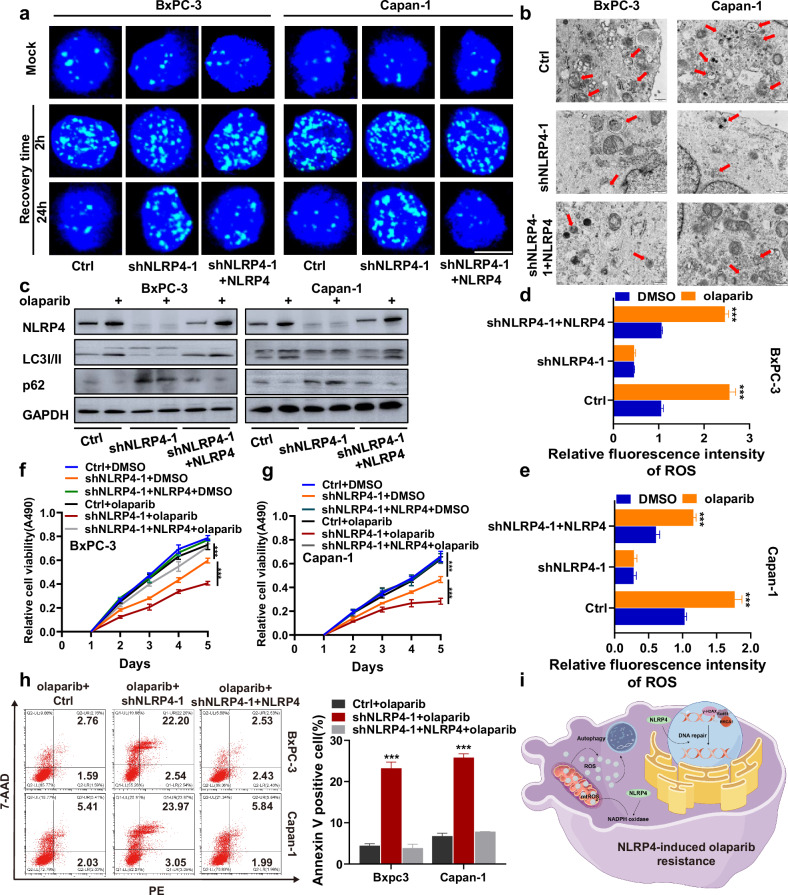
Complementation of NLRP4 rescues DNA repair defects and autophagy levels in NLRP4-knockdown pancreatic cancer cells and results in olaparib resistance. **a** Representative immunofluorescence micrographs of γ-H2AX foci in the indicated cells with or without olaparib treatment (5 μM olaparib for BxPC-3 cells and 500 nM olaparib for Capan-1 cells). **b** TEM-based ultrastructure analysis (autophagosomes) in the indicated cells. **c** Cells were treated with DMSO or olaparib (5 μM olaparib for BxPC-3 cells and 500 nM olaparib for Capan-1 cells) for 48 h. Western blotting analysis was performed for the indicated antibodies. **d**, **e** Cells treated with DMSO or olaparib (5 μM olaparib for BxPC-3 cells and 500 nM olaparib for Capan-1 cells) for 48 h were incubated with an ROS indicator, and fluorescence intensity was assessed with flow cytometry. The analysis of significant differences was performed with one-way ANOVA. ***p < 0.001. **f**, **g** The indicated cells were treated with DMSO or olaparib (5 μM olaparib for BxPC-3 cells and 500 nM olaparib for Capan-1 cells). MTS assays were performed to investigate the effect of NLRP4 on olaparib resistance. The analysis of significant differences was performed with one-way ANOVA. ***p < 0.001. **h** Flow cytometry results for annexin V/7-ADD staining in the indicated cell lines after exposure to DMSO or olaparib (5 μM olaparib for BxPC-3 cells and 500 nM olaparib for Capan-1 cells). The analysis of significant differences was performed with Student’s *t* test. ***p < 0.001. **i** Schematic model showing that NLRP4 renders pancreatic cancer resistant to PARPi through the promotion of the DNA damage response and ROS-induced autophagy.

To eliminate the potential off-target effects of NLRP4 shRNA, we generated NLRP4-knockdown BxPC-3 and Capan-1 cell lines with NLRP4 complementation (Extended Data Fig. 9a). Our findings demonstrate that NLRP4 complementation effectively rescues the DNA repair defects induced by NLRP4 knockdown in response to exposure to olaparib (Fig. 9a and Extended Data Fig. 9b–f). Additionally, the reduction in autophagosome formation caused by NLRP4 knockdown was reversed by NLRP4 complementation (Fig. 9b and Extended Data Fig. 9g, h). Moreover, compared to the findings in NLRP4-knockdown cell lines, NLRP4 complementation promoted the transition of LC3I to LC3II (Fig. 9c and Extended Data Fig. 9i, j). Furthermore, the results indicated that the inhibition of ROS induced by NLRP4 knockdown in response to exposure to olaparib could be restored through NLRP4 complementation (Fig. 9d, e and Extended Data Fig. 9k, l). Moreover, the proliferation of pancreatic cancer cells and their resistance to olaparib were found to increase upon NLRP4 complementation when compared to the levels in cells with NLRP4 knockdown (Fig. 9f-g). Olaparib-induced apoptosis in pancreatic cancer cells decreased with NLRP4 complementation compared NLRP4 knockdown (Fig. 9h). These findings provide evidence that NLRP4 plays a role in promoting the DNA damage response and ROS-induced autophagy, thereby conferring resistance to olaparib on pancreatic cancer cells (Fig. 9i).

## Discussion

Olaparib has been granted approval for use for the treatment of metastatic pancreatic cancer with BRCA1/2 mutations. However, a significant proportion of pancreatic cancer patients either have inherent resistance or develop tolerance to olaparib. Understanding the molecular mechanisms underlying olaparib resistance is essential for the advancement of effective targeted therapies for pancreatic cancer. The restoration of the DNA repair pathway is the predominant factor contributing to resistance against PARPi. Additionally, disrupted DNA repair signaling has been identified as a factor contributing to drug resistance in various cancer types, including pancreatic cancer [30–32].

The administration of olaparib led to elevated levels of ROS and intracellular free radicals, potentially inducing autophagy [33–36]. Our study provides evidence that NLRP4 induces intracellular ROS generation in pancreatic cancer cells, thereby promoting autophagy and contributing to olaparib resistance. Mechanistically, olaparib upregulates NLRP4 expression, which in turn inhibits the nuclear localization of Sirt7. This inhibition results in increased acetylation of H3K18 in the promoter region of the NADPH oxidase subunit NOXO1, leading to enhanced NOXO1 transcription. Mitochondria and NADPH oxidase are recognized as primary sources of intracellular ROS [37, 38], and our investigation reveals that NLRP4 enhances ROS generation specifically in mitochondria, contributing to mitochondrial dysfunction.

Recently, the phenomenon known as “ROS-induced ROS release,” which involves the interplay between ROS generated by NADPH oxidase and mitochondria, has been identified as a mechanism underlying the amplification of ROS within different subcellular compartments [37, 39–41]. It is plausible that “ROS-induced ROS release” serves as a feed-forward mechanism for the intracellular ROS induced by NLRP4. The inhibition of mitochondrial ROS using MitoQ resulted in the suppression of NLRP4-induced autophagic flux. Furthermore, the levels of intracellular ROS in NLRP4-knockdown pancreatic cancer cells and control cells treated with MitoQ remained unchanged, suggesting that NLRP4 triggers autophagy through ROS signaling. Intriguingly, an investigation was conducted on the γ-H2AX foci under various cellular conditions, including control, NLRP4 knockdown, NLRP4 overexpression in pancreatic cancer cells, and MitoQ treatment, after olaparib treatment. The consistent γ-H2AX levels observed in these cells suggest that NLRP4-induced DNA repair may not rely on mitochondrial ROS.

Recent research has highlighted the anticancer and drug-resistant properties of autophagy [39, 42, 43]. This study aimed to investigate the impact of autophagy on the sensitivity of pancreatic cancer cells to olaparib. Moderate autophagy plays a crucial role in eliminating excessive ROS, thereby safeguarding cells against oxidative harm. Conversely, inhibiting autophagy may render cancer cells more vulnerable to anticancer therapies, as it serves as a protective mechanism during treatment. Activation of the DNA damage response can induce autophagy, which represents one of the DNA damage tolerance mechanisms within the DDR framework. Therefore, it is postulated that dysregulated autophagy may contribute to the enhancement of olaparib resistance in pancreatic cancer cells, as it serves to safeguard them against cellular damage. Numerous studies have demonstrated that ROS promote DNA damage and induce autophagy [44, 45]. Autophagy, in turn, contributes to maintaining genome stability through its involvement in DNA repair regulation, senescence, and cytokine secretion [45–47]. Our study revealed that NLRP4-induced accumulation of ROS triggers autophagy in cells. However, NLRP4-induced ROS accumulation was insufficient to cause DNA damage. The relationship between NLRP4-induced autophagy and NLRP4-regulated DNA repair remains unexplored. NLRP4 promotes NOXO1 transcription by inhibiting the nuclear translocation of Sirt7, thereby inducing ROS production and autophagy. The RNA-seq results indicate that NLRP4 also promotes the expression of DNA damage repair genes such as BRCA1 and Rad51. Perhaps the upregulation of these genes is similar to the mechanism by which NLRP4 regulates NOXO1.

Indeed, our investigation revealed that the administration of olaparib resulted in a reduction in autophagy in NLRP4-knockdown pancreatic cancer cells, which was correlated with an increased sensitivity to olaparib. These findings suggest that autophagy may function as a protective mechanism during the treatment of pancreatic cancer with olaparib, which is in agreement with prior observations. Consequently, the targeting of autophagy-regulating molecules may offer a potential avenue for overcoming resistance to pancreatic cancer therapy, as cells may employ this mechanism to evade or delay apoptotic cell death induced by olaparib.

NLRP4, a member of the NLR family associated with inflammasomes, has been extensively investigated for its autophagic functions [48, 49]. However, its physiological significance in relation to DNA repair and ROS generation remains unknown. Furthermore, few studies have explored the association between NLRP4 and olaparib resistance in pancreatic cancer. In this study, we conducted in vitro and in vivo evaluations to assess the impact of NLRP4 on olaparib resistance. Our findings indicate that NLRP4 knockdown resulted in increased sensitivity to olaparib compared to that in the control groups. We determined that the mechanism underlying the development of olaparib resistance in pancreatic cancer cells is attributable to elevated levels of ROS and autophagy. Notably, the administration of the autophagy inhibitor CQ or the ROS inhibitor MitoQ prior to treatment significantly enhanced the sensitivity of pancreatic cancer cells to olaparib. Significantly, these findings were observed in both BRCA wild-type and mutant cells, suggesting the broader potential applicability of these therapeutic approaches.

In conclusion, this study demonstrated that NLRP4 enhances DNA repair capability and promotes ROS-induced autophagy under both basal conditions and treatment with olaparib, thereby leading to the development of olaparib resistance in pancreatic cancer cells both in vitro and in vivo. However, further investigation is needed to elucidate the specific mechanisms by which NLRP4 is involved in autophagy and DNA repair. The findings of our research regarding the role of NLRP4 in regulating autophagy and olaparib sensitivity have revealed a distinctive function of NLRP4 in pancreatic cancer, which may have significant implications for determining the most effective treatment approach for pancreatic cancer patients.

### Supplementary information

NLRP4 Extended Data

Original data files

## Data Availability

The transcriptome data generated by the study have been uploaded as bioproject PRJCA026954 of the National Genomics Data Center (NGDC) (https://ngdc.cncb.ac.cn/). The mass spectrometry proteomics data were deposited to the ProteomeXchange Consortium via the iProX partner repository with the dataset identifier PXD051042 (https://www.iprox.cn/page/home.html). All the relevant data that support the findings of this study are available from the corresponding author on request.
