# Supplementary material for: NLRP4 renders pancreatic cancer resistant to olaparib through promotion of the DNA damage response and ROS-induced autophagy
Source: Cell Death Dis. 2024 Aug 26;15(8):620. doi: 10.1038/s41419-024-06984-0 (PMC11347561; doi:10.1038/s41419-024-06984-0)
Supplement: Supplementary file 1 — NLRP4 Extended Data [file 41419_2024_6984_MOESM1_ESM.pdf]

Extended Data

a b c

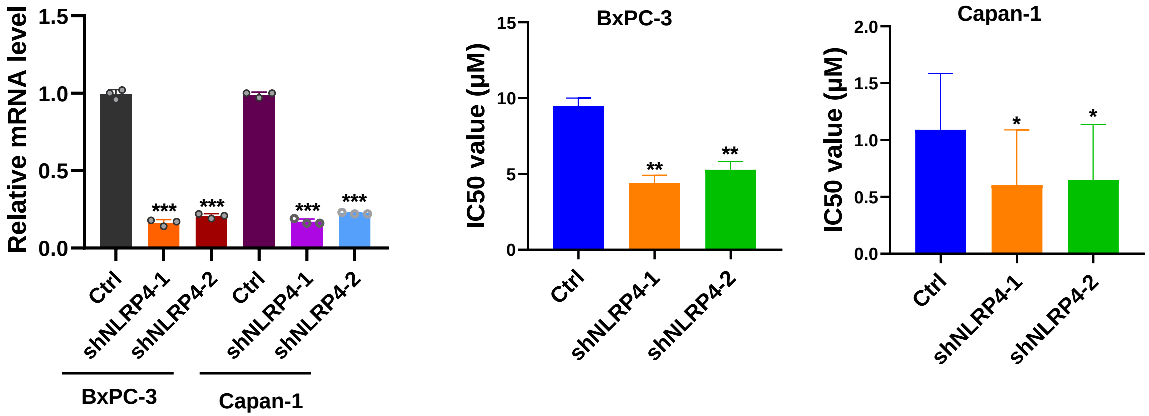

d

BxPC-3	IC50（μM）
Ctrl	9.471±1.194
shNLRP4-1	4.410±1. 151
**Capan-1**	**IC50(μM)**
Ctrl	1.090±1. 109
shNLRP4-1	0.606±1.080
shNLPR4-2	0.648±1.096

e

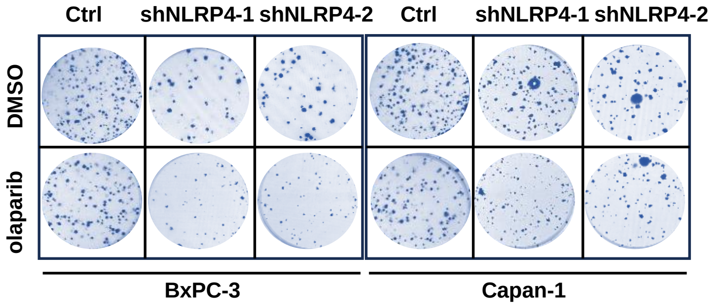

Extended Data Fig. 1

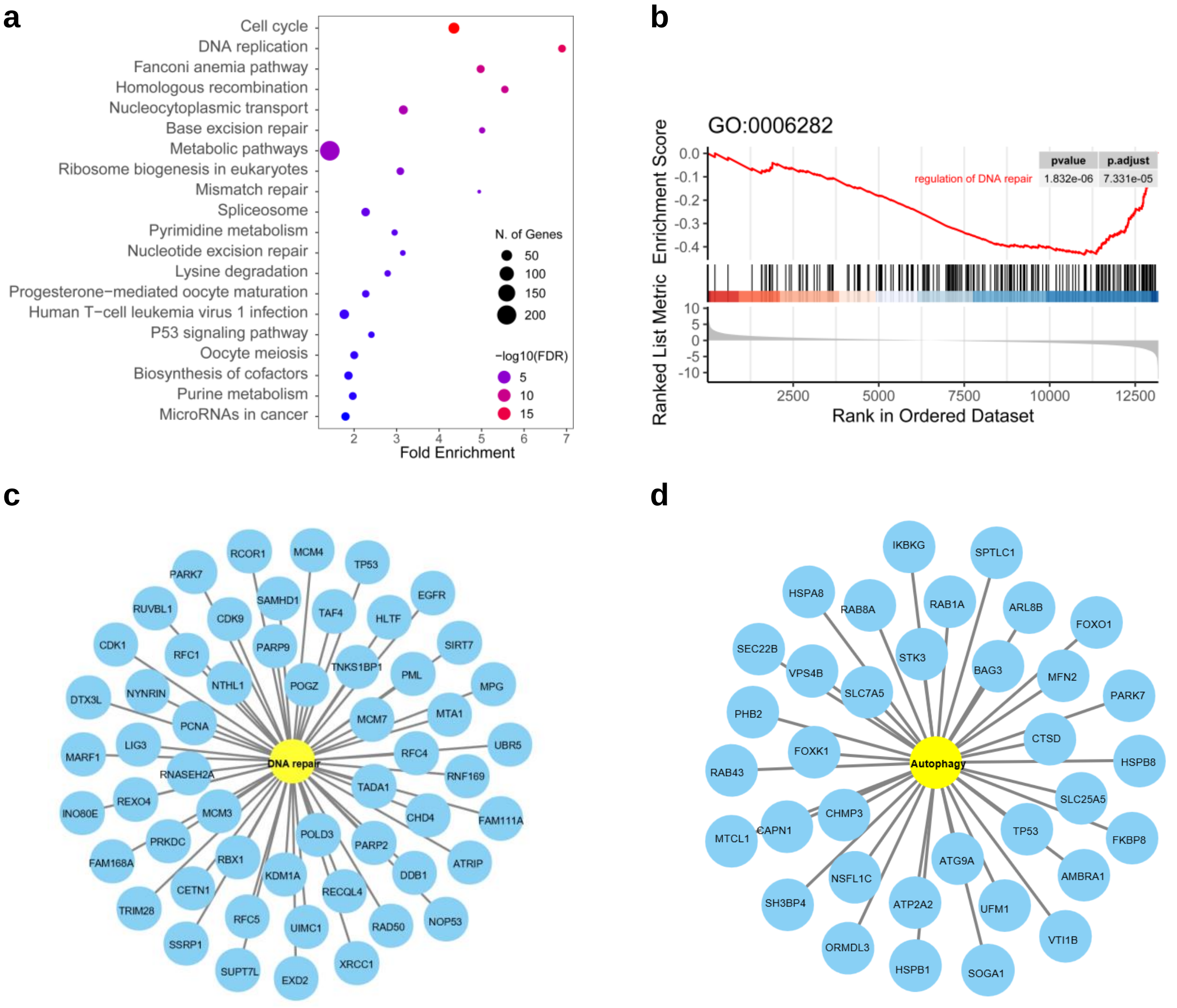

Extended Data Fig. 2

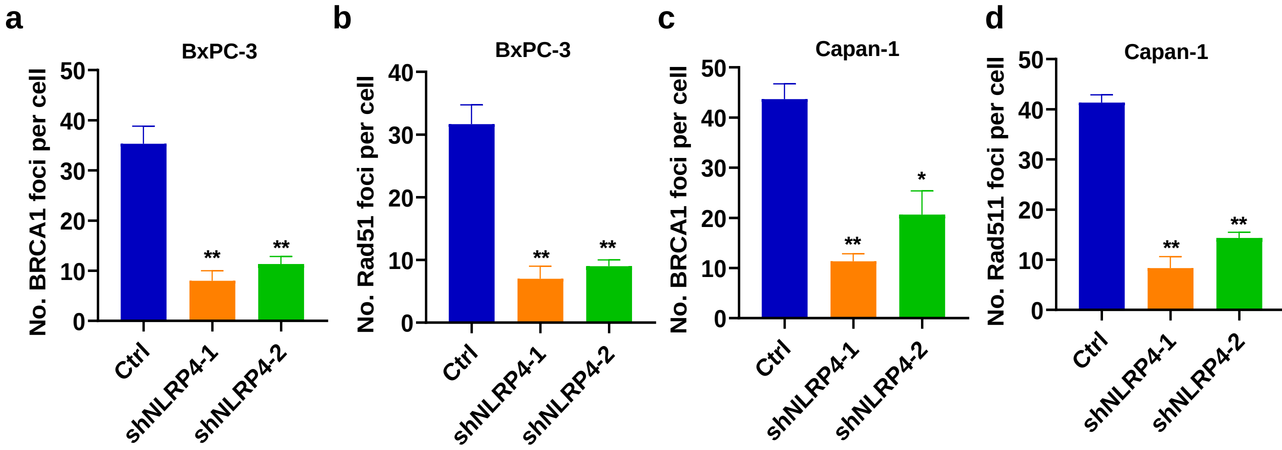

Extended Data Fig. 3

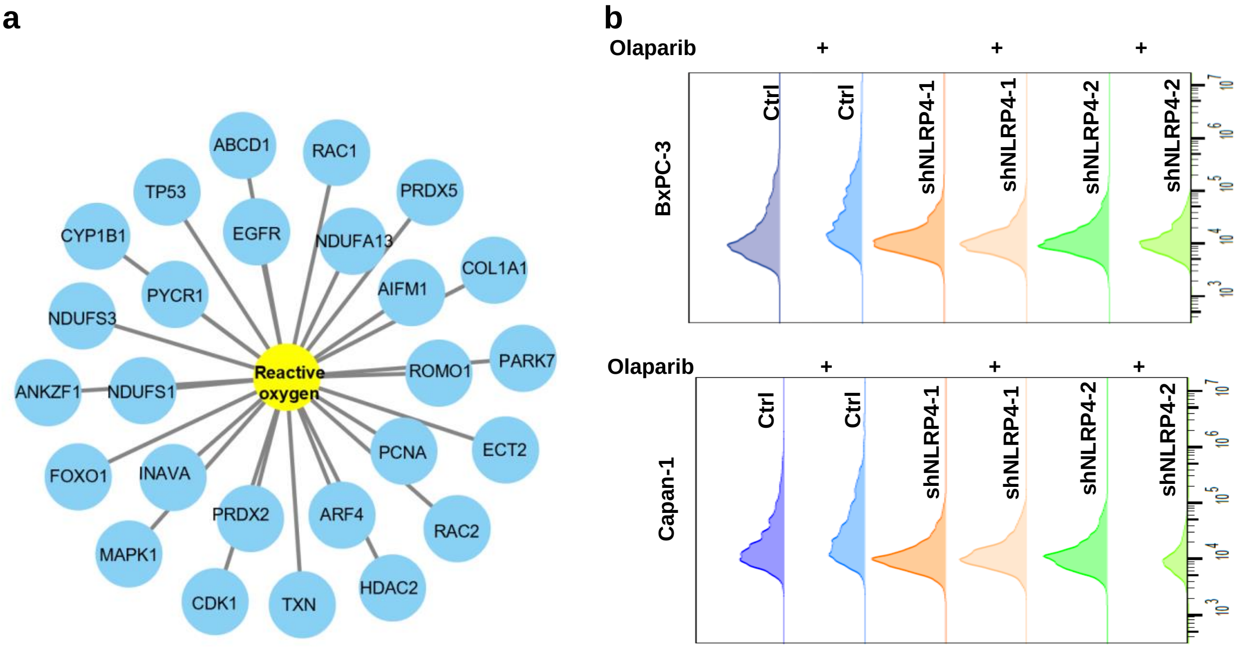

Extended Data Fig. 4

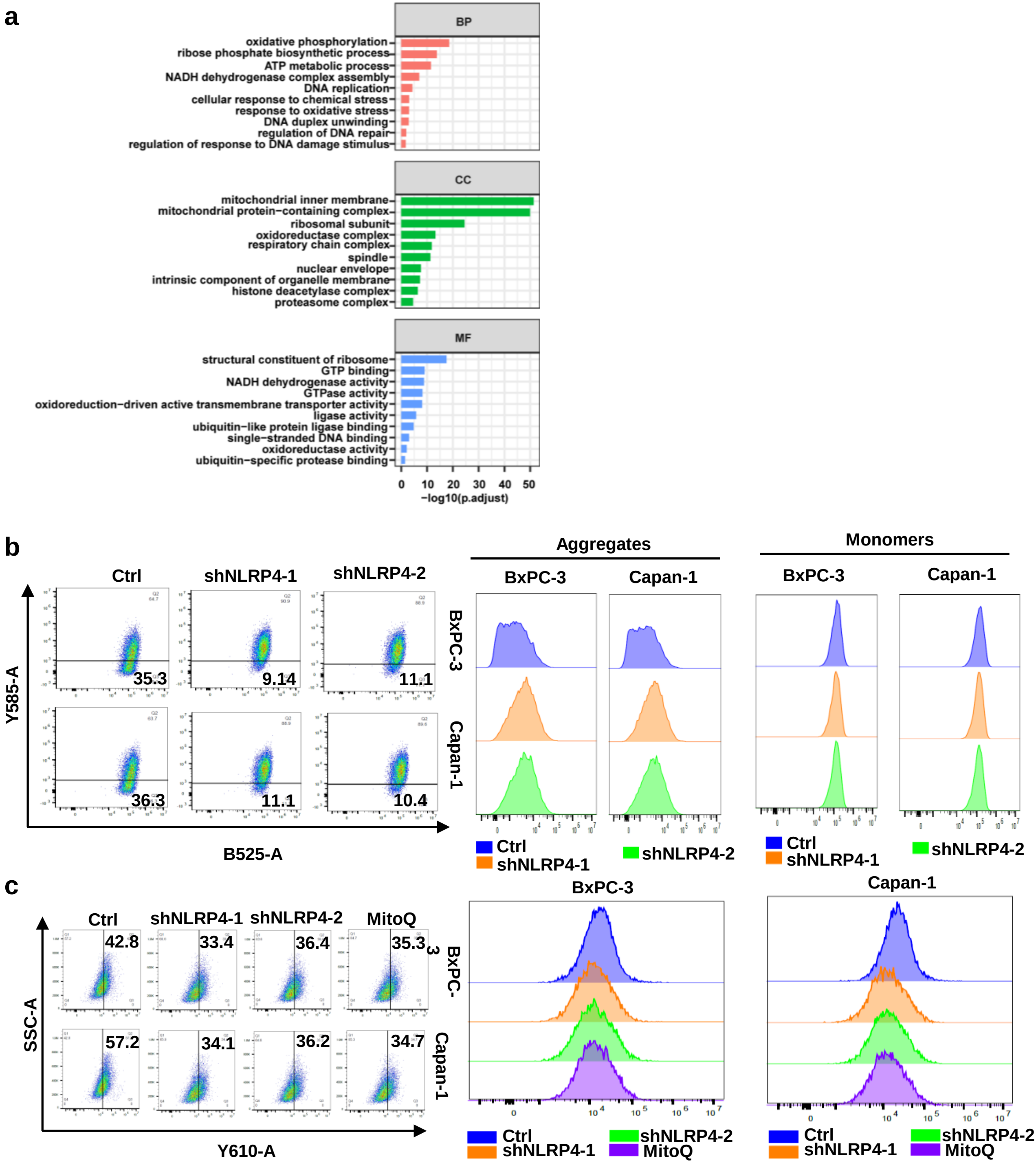

Extended Data Fig. 5

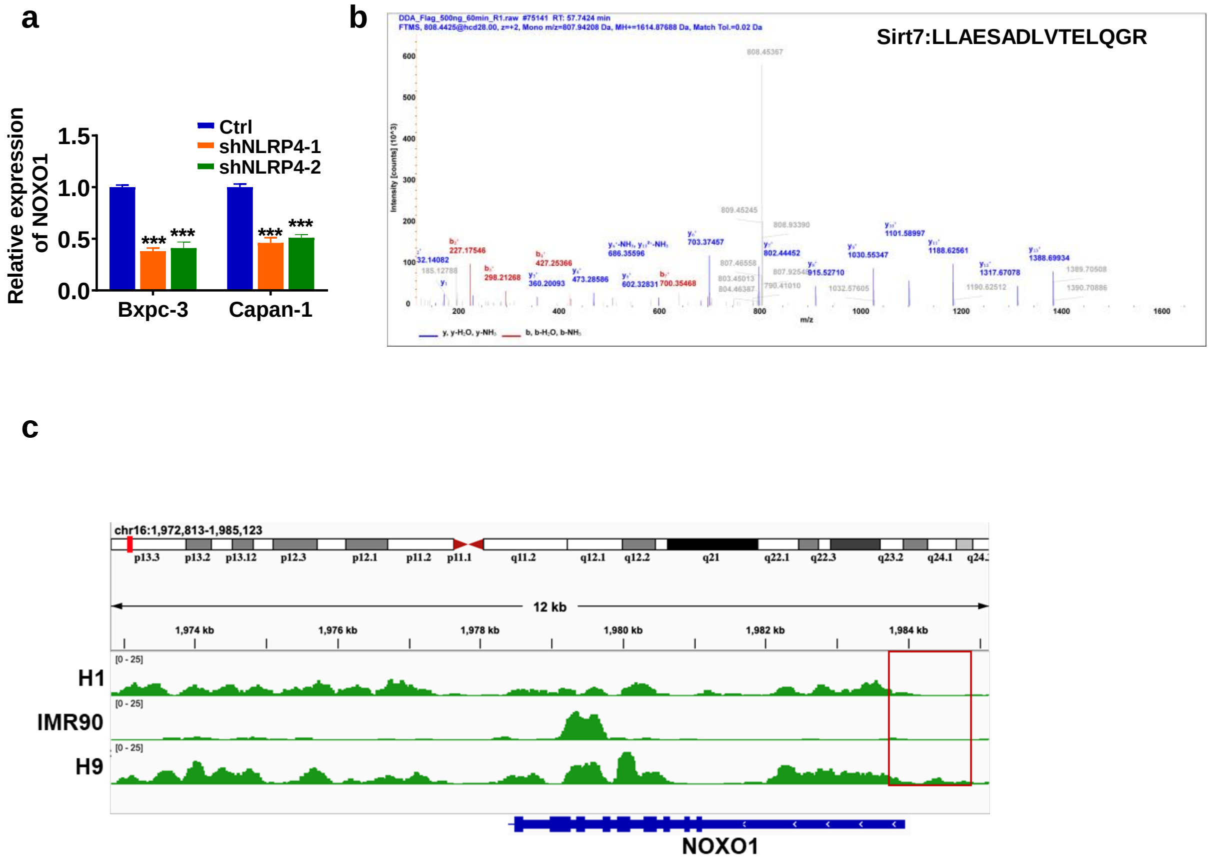

d

	Region	Forward primer Sequence (5'->3')	Reverse primer Sequence (5'->3')
a site	1981469-1981769	GCTAATCAAAGCGCCACCTG	GAAATCCCCAGGCCAGTTCA
b site	1981769-1982069	TATACATGCTGGGGGAGGGG	AGATGGGCCCTCTTTCTCCT
c site	1982069-1982369	AGGGAGCAGTCAGCCTCTAA	GCCCTATGTGAGCAGGTGTT
d site	1982369-1982669	GGAAGACAGGAGAAGCTGGG	GGGGCAAGTGAGGAGCATAC
e site	1982669-1982969	CCGCTGGCTATCATATGGGG	CTGCAGTGAACAAGATCGCG
f site	1982969-1983269	CCTTGCCCAAAACTGTCTAGC	ACCAAAGACTTGGTCGGCAG
g site	1983269-1983569	CTTGCATGGACCCACCAGTT	CAGTGACCGCTCTGTTCCAA
h site	1983569-1983869	CGTTGAGAGAGCGCTTGGC	GGCAAAGTCCGTGTTGCG
i site	1983869-1984169	TTTACTGGAGAAAGCGGGACG	CCTCAGGCGTCGATCCG
j site	1984169-1984469	GAGATGATGGACGACCTGGC	ACAGGAGAATCGGAGGTCGG
k site	1984469-1984769	GGCCTTACAGCCTTCATCCG	CCCTAAACTTGGTGTCCCGC

Extended Data Fig. 6

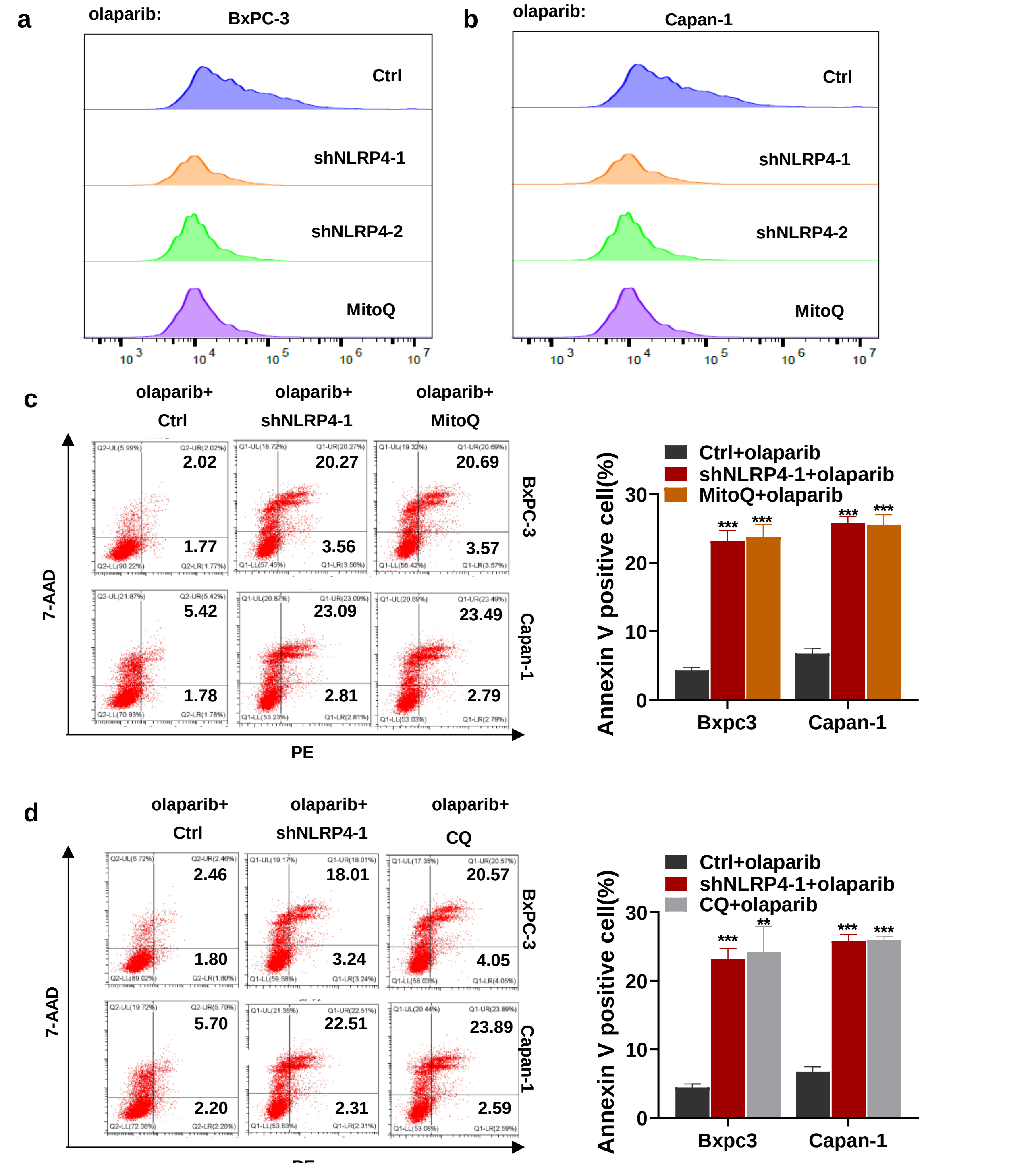

Extended Data Fig. 7

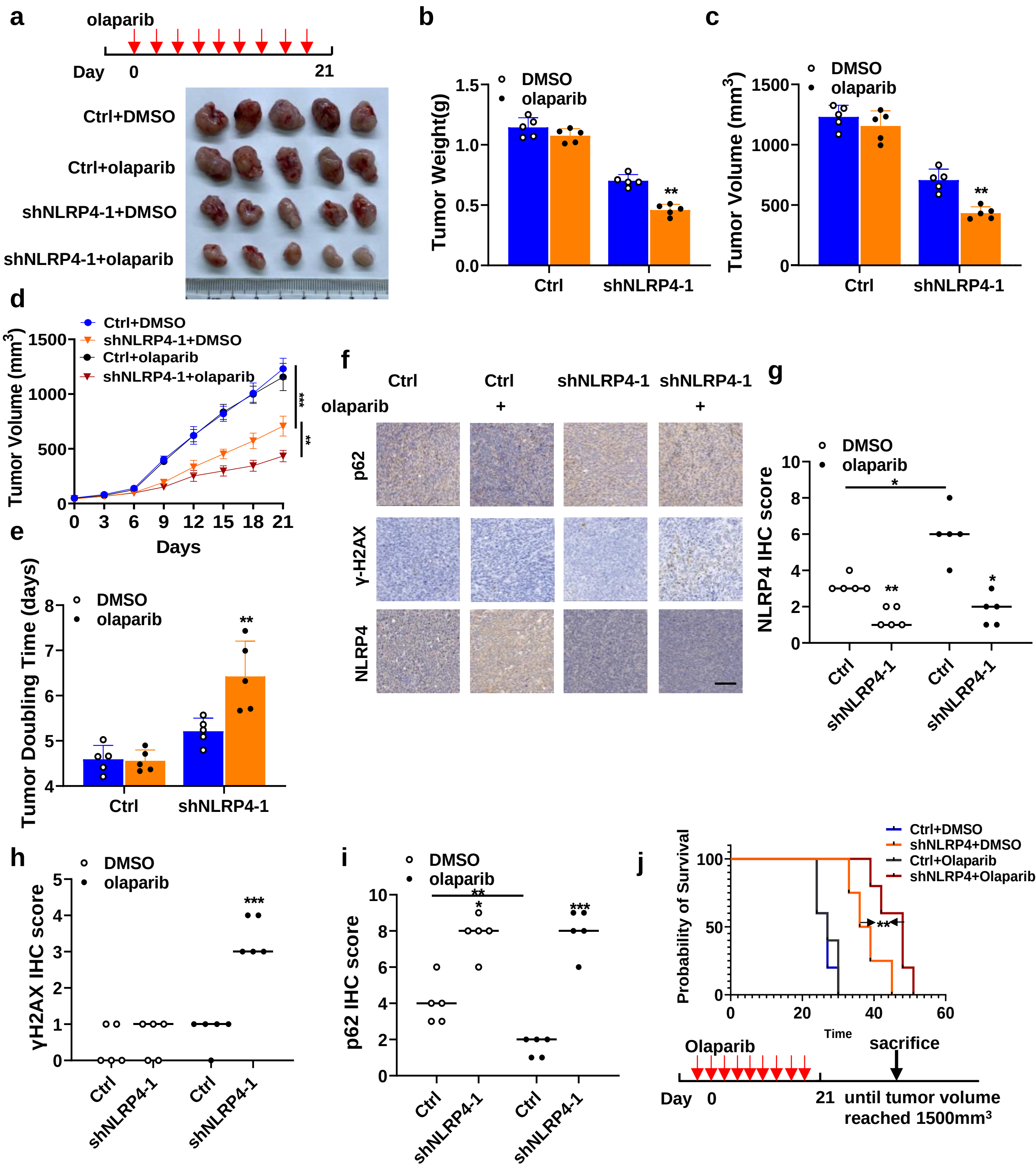

Extended Data Fig. 8

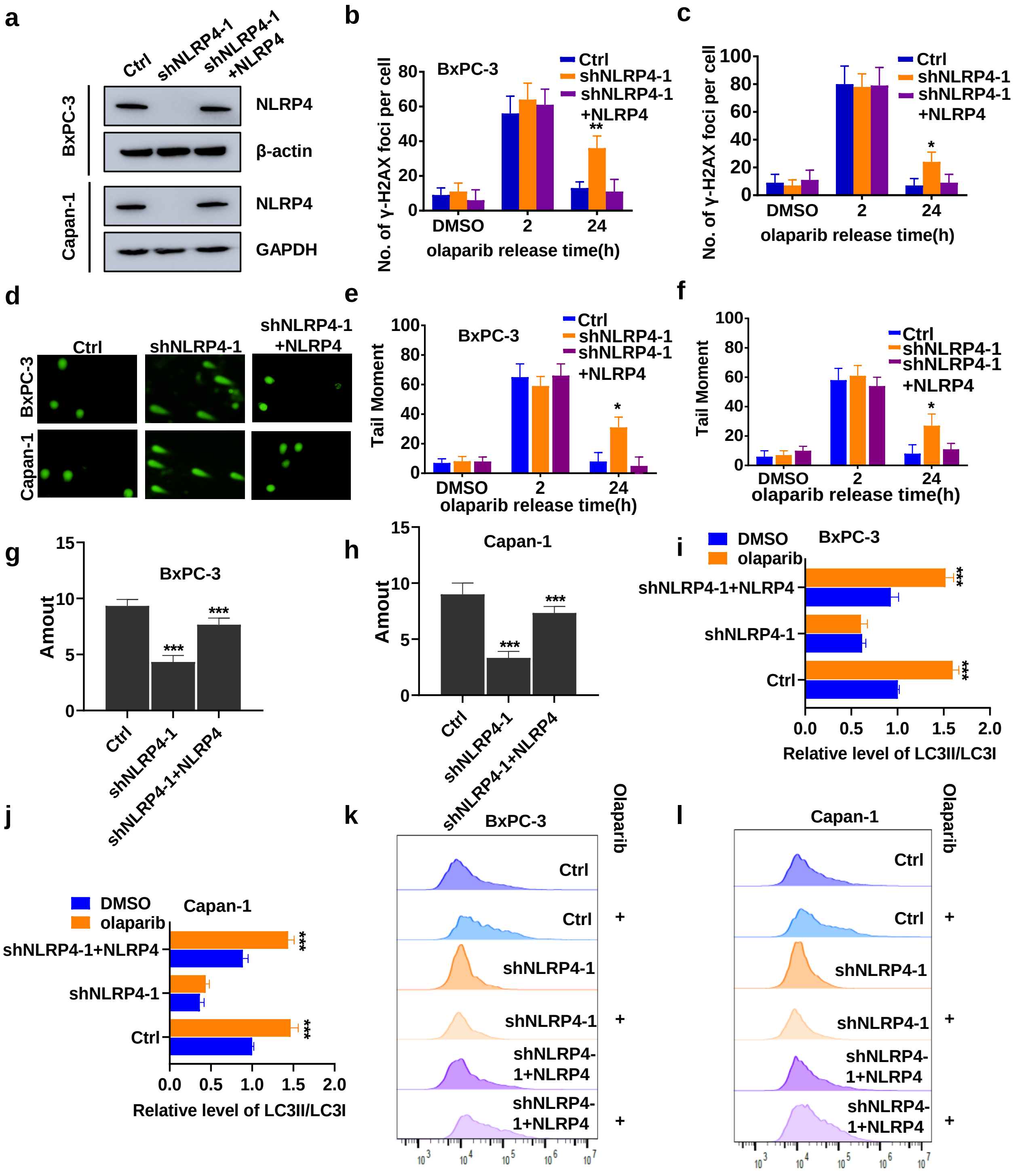

Extended Data Fig. 9

Extended Data Figure Legends

Extended Data Fig.1 (a) Cells were harvested, and RNA was extracted for RT–qPCR analysis to determine NLRP4 expression. Data are shown as the mean ± SD (n=3). ***, $P < 0.001$. (b-d) MTS assays were performed to calculate the IC50 of olaparib in the indicated cells. *, $P < 0.05$. The analysis of significant differences was performed with Student's t test. (e) Representative images of the colony formation assay.

Extended Data Fig.2 (a) Control or NLRP4-knockdown Capan-1 cells were treated with DMSO or olaparib (500 nM) for RNA sequencing and were subjected to KEGG pathway enrichment analysis. (b) GO pathway enrichment analysis of DNA repair. (c-d) The NLRP4 reactome pathway was implicated in the DNA damage response (c) and autophagy pathway (d), as revealed by NLRP4 Co-IP MS analysis.

Extended Data Fig.3 (a-d) Quantification of BRCA1 or Rad51 foci in the indicated cell lines at 6 h after olaparib treatment. *, $P < 0.05$, **, $P < 0.01$. The analysis of significant differences was performed with Student's t test.

Extended Data Fig.4 (a) NLRP4-related ROS reactome pathway by Co-IP MS analysis. (b) Cells treated with DMSO or olaparib (5 μM olaparib for BxPC-3 cells and 500 nM olaparib for Capan-1 cells) for 48 h were incubated with an ROS indicator, and fluorescence intensity was assessed with flow cytometry. The analysis of significant differences was performed with Student's t test. ***, $P < 0.001$.

Extended Data Fig.5 (a) NLRP4-related GO pathway by Co-IP MS analysis. (b) Cells treated with DMSO or olaparib (5 μM olaparib for BxPC-3 cells and 500 nM olaparib for Capan-1 cells) for 48 h were incubated with JC-1 working solution, and fluorescence intensity was assessed with flow cytometry. (c) Cells treated with olaparib (5 μM olaparib for BxPC-3 cells and 500 nM olaparib for Capan-1 cells) plus DMSO or MitoQ for 48 h were incubated with MitoSOX. Representative images were obtained,

and the fluorescence intensity was assessed with flow cytometry.

Extended Data Fig.6 (a) qPCR analysis of NOXO1 expression. (b) Sirt7 was identified by LC-MS. (c) Bioinformatics analysis filtered NOXO1 as a downstream target of H3K18ac. (d) Primers for ChIP-qPCR.

Extended Data Fig.7 (a-b) Cells treated with olaparib (5 μM olaparib for BxPC-3 cells and 500 nM olaparib for Capan-1 cells) plus DMSO or MitoQ for 48 h were incubated with an ROS indicator, and fluorescence intensity was assessed with flow cytometry. (c) The indicated cells were treated with DMSO or olaparib (5 μM olaparib for BxPC-3 cells and 500 nM olaparib for Capan-1 cells) with or without MitoQ. Flow cytometry analysis for annexin V/7-ADD staining was performed. The analysis of significant differences was performed with Student's t test. ***, $P < 0.001$.(d) The indicated cells were treated with DMSO or olaparib (5 μM olaparib for BxPC-3 cells and 500 nM olaparib for Capan-1 cells) with or without CQ. Flow cytometry analysis for annexin V/7-ADD staining was performed. The analysis of significant differences was performed with Student's t test. ***, $P < 0.001$. **, $P< 0.01$.

Extended Data Fig.8 (a) Capan-1 xenografts obtained from mice in different groups treated with DMSO or olaparib (50 mg/kg per day). (b) Quantification of the weight of the tumors in different groups treated with DMSO or olaparib (50 mg/kg per day). The analysis of significant differences was performed with Student's t test. (c) Quantification of tumor volume on the last day in different groups treated with DMSO or olaparib (50 mg/kg per day). The analysis of significant differences was performed with Student's t test. (d) Growth curves of cells treated with DMSO or olaparib. The analysis of significant differences was performed with one-way ANOVA. (e) Tumor doubling time in different groups treated with DMSO or olaparib (50 mg/kg per day). The analysis of significant differences was performed with Student's t test. (f-h) Tumors

were subjected to immunological staining to detect the indicated marker. (f) Representative IHC micrographs. Scale bar, 100 μm. (g-i) The histological score (H score) of the indicated markers was quantified. The analysis of significant differences was performed with Student's t test. (j) Kaplan–Meier survival curves for the indicated groups.

Extended Data Fig.9 (a) Immunoblot of NLRP4 in the indicated BxPC-3 and Capan-1 cells. (b-c) Quantification of γ-H2AX foci in the indicated cells with or without olaparib treatment (5 μM olaparib for BxPC-3 cells and 500 nM olaparib for Capan-1 cells). The analysis of significant differences was performed with Student's t test. **, $P < 0.01$, *, $P < 0.05$. Scale bar 5 μm. (d-f) Representative comet assay micrographs and quantification of tail moments in the indicated cells with or without olaparib treatment (5 μM olaparib for BxPC-3 cells and 500 nM olaparib for Capan-1 cells). The analysis of significant differences was performed with Student's t test. *, $P < 0.05$. (g-h) TEM-based ultrastructure analysis (autophagosomes) in the indicated cells. The analysis of significant differences was performed with Student's t test. ***, $P < 0.001$. (i-j) Quantification of LC3II/LC3I. (k-l) Cells treated with DMSO or olaparib (5 μM olaparib for BxPC-3 cells and 500 nM olaparib for Capan-1 cells) for 48 h were incubated with an ROS indicator, and fluorescence intensity was assessed with flow cytometry.
