# Supplementary material for: NLRP4 renders pancreatic cancer resistant to olaparib through promotion of the DNA damage response and ROS-induced autophagy
Source: Cell Death Dis. 2024 Aug 26;15(8):620. doi: 10.1038/s41419-024-06984-0 (PMC11347561; doi:10.1038/s41419-024-06984-0)

Fig 6g

	Sample Name	Target Name	C_T
**BxPC-3**	Input	a site	23.03
	Input	a site	24.2
	Input	a site	25.62
	H3K18ac	a site	22.48
	H3K18ac	a site	21.71
	H3K18ac	a site	21.82
	IgG	a site	27.71
	IgG	a site	27.38
	IgG	a site	27.33
	Input	b site	25.38
	Input	b site	25.01
	Input	b site	24.51
	H3K18ac	b site	27.89
	H3K18ac	b site	27.38
	H3K18ac	b site	27.07
	IgG	b site	27.06
	IgG	b site	27.19
	IgG	b site	26.83
	Input	c site	23.77
	Input	c site	23.88
	Input	c site	22.59
	H3K18ac	c site	26.01
	H3K18ac	c site	26.39
	H3K18ac	c site	26.06
	IgG	c site	26.06
	IgG	c site	25.72
	IgG	c site	25.96
	Input	d site	22.72
	Input	d site	23.12
	Input	d site	23.7
	H3K18ac	d site	24.06
	H3K18ac	d site	24.9
	H3K18ac	d site	24.89
	IgG	d site	24.81
	IgG	d site	24.56
	IgG	d site	25.43
	Input	e site	23.23
	Input	e site	23.34
	Input	e site	21.95
	H3K18ac	e site	24.05
	H3K18ac	e site	24.17
	H3K18ac	e site	24.91
	IgG	e site	25.04
	IgG	e site	24.66
	IgG	e site	24.82
	Input	f site	22.86
	Input	f site	23.04
	Input	f site	22.4
	H3K18ac	f site	25.24
	H3K18ac	f site	24.8
	H3K18ac	f site	25.05

IgG	f site	25.41
IgG	f site	25.76
IgG	f site	25.83
Input	g site	24.36
Input	g site	24.28
Input	g site	23.38
H3K18ac	g site	25.99
H3K18ac	g site	25.47
H3K18ac	g site	26.19
IgG	g site	27.35
IgG	g site	26.49
IgG	g site	26.85
Input	h site	22.9
Input	h site	22.43
Input	h site	21.75
H3K18ac	h site	25.38
H3K18ac	h site	25.35
H3K18ac	h site	25.21
IgG	h site	23.96
IgG	h site	24.5
IgG	h site	23.86
Input	i site	24.66
Input	i site	24.6
Input	i site	24.47
H3K18ac	i site	27.16
H3K18ac	i site	26.2
H3K18ac	i site	26.84
IgG	i site	26.87
IgG	i site	26.51
IgG	i site	26.01
Input	j site	26.34
Input	j site	25.3
Input	j site	26.03
H3K18ac	j site	28.2
H3K18ac	j site	28.77
H3K18ac	j site	28.32
IgG	j site	28.87
IgG	j site	28.72
IgG	j site	28.32
Input	k site	22.6
Input	k site	22.12
Input	k site	22.27
H3K18ac	k site	23.29
H3K18ac	k site	23.95
H3K18ac	k site	23.25
IgG	k site	24.58
IgG	k site	24.23
IgG	k site	23.87

Fig 6h

Capan-1	Sample Name	Target Name	Cт
	Input	a site	25.29
	Input	a site	24.95
	Input	a site	25.35
	H3K18ac	a site	23.48
	H3K18ac	a site	22.92
	H3K18ac	a site	23.4
	IgG	a site	27.95
	IgG	a site	27.97
	IgG	a site	27.69
	Input	b site	24.1
	Input	b site	24.51
	Input	b site	25.27
	H3K18ac	b site	27.29
	H3K18ac	b site	27.38
	H3K18ac	b site	26.39
	IgG	b site	26.58
	IgG	b site	27.05
	IgG	b site	26.4
	Input	c site	24.78
	Input	c site	24.99
	Input	c site	24.8
	H3K18ac	c site	26.86
	H3K18ac	c site	26.09
	H3K18ac	c site	26.21
	IgG	c site	27.69
	IgG	c site	27.71
	IgG	c site	27.13
	Input	d site	25.06
	Input	d site	24.47
	Input	d site	24.69
	H3K18ac	d site	26.72
	H3K18ac	d site	27.42
	H3K18ac	d site	27.54
	IgG	d site	26.87
	IgG	d site	26.2
	IgG	d site	25.59
	Input	e site	26.53
	Input	e site	25.29
	Input	e site	25.44
	H3K18ac	e site	28.01
	H3K18ac	e site	28.76
	H3K18ac	e site	28.48
	IgG	e site	26.96
	IgG	e site	27.09
	IgG	e site	27.41
	Input	f site	25.21
	Input	f site	25.09
	Input	f site	25.69
	H3K18ac	f site	27.07
	H3K18ac	f site	27.71
	H3K18ac	f site	26.74

IgG	f site	27.42
IgG	f site	27.96
IgG	f site	28.16
Input	g site	25.27
Input	g site	25.11
Input	g site	25.52
H3K18ac	g site	27.33
H3K18ac	g site	26.68
H3K18ac	g site	27
IgG	g site	27.58
IgG	g site	27.44
IgG	g site	27.61
Input	h site	24.94
Input	h site	24.59
Input	h site	25.08
H3K18ac	h site	27.72
H3K18ac	h site	26.83
H3K18ac	h site	26.59
IgG	h site	27.99
IgG	h site	27.33
IgG	h site	27.24
Input	i site	24.95
Input	i site	24.98
Input	i site	25.39
H3K18ac	i site	26.72
H3K18ac	i site	27.12
H3K18ac	i site	26.62
IgG	i site	27.68
IgG	i site	27.11
IgG	i site	27.01
Input	j site	24.6
Input	j site	24.38
Input	j site	23.98
H3K18ac	j site	26.2
H3K18ac	j site	26.19
H3K18ac	j site	25.49
IgG	j site	26.78
IgG	j site	26.78
IgG	j site	26.54
Input	k site	24.62
Input	k site	24.72
Input	k site	23.95
H3K18ac	k site	24.37
H3K18ac	k site	23.09
H3K18ac	k site	22.64
IgG	k site	22.97
IgG	k site	23.44
IgG	k site	22.74

Fig 6i

	Sample Name	Target Name	Cт
**BxPC-3**	Input	a site	23.43
**BxPC-3**	Input	a site	23.9
**BxPC-3**	Input	a site	24.61
**BxPC-3**	ctrl	a site	21.21
**BxPC-3**	ctrl	a site	21.71
**BxPC-3**	ctrl	a site	22.21
**BxPC-3**	shNLRP4-1	a site	23.25
**BxPC-3**	shNLRP4-1	a site	23.46
**BxPC-3**	shNLRP4-1	a site	22.89
**BxPC-3**	shNLRP4-2	a site	23.1
**BxPC-3**	shNLRP4-2	a site	22.51
**BxPC-3**	shNLRP4-2	a site	23.27
**Capan-1**	Input	a site	25.57
**Capan-1**	Input	a site	25.22
**Capan-1**	Input	a site	24.63
**Capan-1**	ctrl	a site	23.1
**Capan-1**	ctrl	a site	22.91
**Capan-1**	ctrl	a site	23.59
**Capan-1**	shNLRP4-1	a site	24.81
**Capan-1**	shNLRP4-1	a site	24.85
**Capan-1**	shNLRP4-1	a site	23.69
**Capan-1**	shNLRP4-2	a site	23.97
**Capan-1**	shNLRP4-2	a site	24.49
**Capan-1**	shNLRP4-2	a site	24.46

Fig 6j

	treat 1	treat 2	Target Name	Cт
**BxPC-3**	NC	Input	a site	23.67
**BxPC-3**	NC	Input	a site	24.22
**BxPC-3**	NC	Input	a site	24.28
**BxPC-3**	NC	shNC	a site	21.69
**BxPC-3**	NC	shNC	a site	21.18
**BxPC-3**	NC	shNC	a site	21.93
**BxPC-3**	NC	shSirt7-1	a site	21.47
**BxPC-3**	NC	shSirt7-1	a site	20.69
**BxPC-3**	NC	shSirt7-1	a site	21.06
**BxPC-3**	NC	shSirt7-2	a site	21.2
**BxPC-3**	NC	shSirt7-2	a site	20.75
**BxPC-3**	NC	shSirt7-2	a site	21.45
**BxPC-3**	shNLRP4-1	Input	a site	23.98
**BxPC-3**	shNLRP4-1	Input	a site	23.32
**BxPC-3**	shNLRP4-1	Input	a site	23.91
**BxPC-3**	shNLRP4-1	shNC	a site	22.23
**BxPC-3**	shNLRP4-1	shNC	a site	22.22
**BxPC-3**	shNLRP4-1	shNC	a site	23.73
**BxPC-3**	shNLRP4-1	shSirt7-1	a site	20.81
**BxPC-3**	shNLRP4-1	shSirt7-1	a site	20.45
**BxPC-3**	shNLRP4-1	shSirt7-1	a site	21.02
**BxPC-3**	shNLRP4-1	shSirt7-2	a site	20.98
**BxPC-3**	shNLRP4-1	shSirt7-2	a site	21.23
**BxPC-3**	shNLRP4-1	shSirt7-2	a site	20.46

Fig 6k

	treat 1	treat 2	Target Name	Cт
**Capan-1**	NC	Input	a site	25.89
**Capan-1**	NC	Input	a site	25.25
**Capan-1**	NC	Input	a site	24.1
**Capan-1**	NC	shNC	a site	22.87
**Capan-1**	NC	shNC	a site	23.25
**Capan-1**	NC	shNC	a site	22.69
**Capan-1**	NC	shSirt7-1	a site	21.89
**Capan-1**	NC	shSirt7-1	a site	22.45
**Capan-1**	NC	shSirt7-1	a site	22.34
**Capan-1**	NC	shSirt7-2	a site	22.37
**Capan-1**	NC	shSirt7-2	a site	21.93
**Capan-1**	NC	shSirt7-2	a site	22.05
**Capan-1**	shNLRP4-1	Input	a site	24.98
**Capan-1**	shNLRP4-1	Input	a site	24.32
**Capan-1**	shNLRP4-1	Input	a site	24.39
**Capan-1**	shNLRP4-1	shNC	a site	23.97
**Capan-1**	shNLRP4-1	shNC	a site	23.45
**Capan-1**	shNLRP4-1	shNC	a site	23.63
**Capan-1**	shNLRP4-1	shSirt7-1	a site	21.91
**Capan-1**	shNLRP4-1	shSirt7-1	a site	21.96
**Capan-1**	shNLRP4-1	shSirt7-1	a site	21.32
**Capan-1**	shNLRP4-1	shSirt7-2	a site	22.18
**Capan-1**	shNLRP4-1	shSirt7-2	a site	21.83
**Capan-1**	shNLRP4-1	shSirt7-2	a site	21.05

Fig 6l

	treat 1	treat 2	Target Name	Cт
**BxPC-3**	DMSO	Input	a site	23.67
**BxPC-3**	DMSO	Input	a site	24.53
**BxPC-3**	DMSO	Input	a site	24.12
**BxPC-3**	DMSO	H3K18ac	a site	21.34
**BxPC-3**	DMSO	H3K18ac	a site	21.9
**BxPC-3**	DMSO	H3K18ac	a site	21.61
**BxPC-3**	DMSO	IgG	a site	27.62
**BxPC-3**	DMSO	IgG	a site	27.02
**BxPC-3**	DMSO	IgG	a site	27.28
**BxPC-3**	Olaparib	Input	a site	23.92
**BxPC-3**	Olaparib	Input	a site	24.28
**BxPC-3**	Olaparib	Input	a site	23.71
**BxPC-3**	Olaparib	H3K18ac	a site	21.46
**BxPC-3**	Olaparib	H3K18ac	a site	21.13
**BxPC-3**	Olaparib	H3K18ac	a site	20.48
**BxPC-3**	Olaparib	IgG	a site	27.21
**BxPC-3**	Olaparib	IgG	a site	26.88
**BxPC-3**	Olaparib	IgG	a site	26.52
**Capan-1**	DMSO	Input	a site	23.97
**Capan-1**	DMSO	Input	a site	23.78
**Capan-1**	DMSO	Input	a site	23.47
**Capan-1**	DMSO	H3K18ac	a site	22.03
**Capan-1**	DMSO	H3K18ac	a site	21.62
**Capan-1**	DMSO	H3K18ac	a site	21.34
**Capan-1**	DMSO	IgG	a site	27.47
**Capan-1**	DMSO	IgG	a site	27.23
**Capan-1**	DMSO	IgG	a site	26.86
**Capan-1**	Olaparib	Input	a site	24.6
**Capan-1**	Olaparib	Input	a site	24.54
**Capan-1**	Olaparib	Input	a site	24.3
**Capan-1**	Olaparib	H3K18ac	a site	22.98
**Capan-1**	Olaparib	H3K18ac	a site	21.25
**Capan-1**	Olaparib	H3K18ac	a site	21.31
**Capan-1**	Olaparib	IgG	a site	27.92
**Capan-1**	Olaparib	IgG	a site	27.21
**Capan-1**	Olaparib	IgG	a site	26.63

Fig 6m

	Sample Name	Target Name	Cт
**Bxpc-3**	DMSO	NLRP4	23.08
	DMSO	NLRP4	23.26
	DMSO	NLRP4	22.83
	DMSO	GAP	15.6
	DMSO	GAP	16.25
	DMSO	GAP	16.36
	Olaparib	NLRP4	21.24
	Olaparib	NLRP4	21.47
	Olaparib	NLRP4	21.02
	Olaparib	GAP	15.94
	Olaparib	GAP	16.42
	Olaparib	GAP	16.15
**Capan-1**	DMSO	NLRP4	23.57
	DMSO	NLRP4	23.44
	DMSO	NLRP4	23.78
	DMSO	GAP	18.92
	DMSO	GAP	18.48
	DMSO	GAP	17.54
	Olaparib	NLRP4	21.93
	Olaparib	NLRP4	22.65
	Olaparib	NLRP4	22
	Olaparib	GAP	18.2
	Olaparib	GAP	18.03
	Olaparib	GAP	17.97

Extended Fig.6a

	Sample Name	Target Name	CT
**Bxpc-3**	Ctrl	NOXO1	22.94
	Ctrl	NOXO1	22.26
	Ctrl	NOXO1	23.08
	Ctrl	GAP	15.82
	Ctrl	GAP	15.92
	Ctrl	GAP	16.68
	shNLRP4-1	NOXO1	24.25
	shNLRP4-1	NOXO1	24.32
	shNLRP4-1	NOXO1	23.43
	shNLRP4-1	GAP	16.43
	shNLRP4-1	GAP	15.93
	shNLRP4-1	GAP	15.56
	shNLRP4-2	NOXO1	21.14
	shNLRP4-2	NOXO1	21.87
	shNLRP4-2	NOXO1	21.43
	shNLRP4-2	GAP	16.43
	shNLRP4-2	GAP	16.12
	shNLRP4-2	GAP	15.86
**Capan-1**	Ctrl	NOXO1	23.86
	Ctrl	NOXO1	23.25
	Ctrl	NOXO1	23.69
	Ctrl	GAP	17.29
	Ctrl	GAP	17.17
	Ctrl	GAP	17.36
	shNLRP4-1	NOXO1	24.73
	shNLRP4-1	NOXO1	25.12
	shNLRP4-1	NOXO1	25.41
	shNLRP4-1	GAP	17.42
	shNLRP4-1	GAP	17.53
	shNLRP4-1	GAP	17.97
	shNLRP4-2	NOXO1	22.52
	shNLRP4-2	NOXO1	22.51
	shNLRP4-2	NOXO1	22.94
	shNLRP4-2	GAP	17.42
	shNLRP4-2	GAP	17.32
	shNLRP4-2	GAP	17.12

Unprocessed western blots

Fig 1b

β-actin NLRP4

BxPC-3

Capan-1

β-actin NLRP4

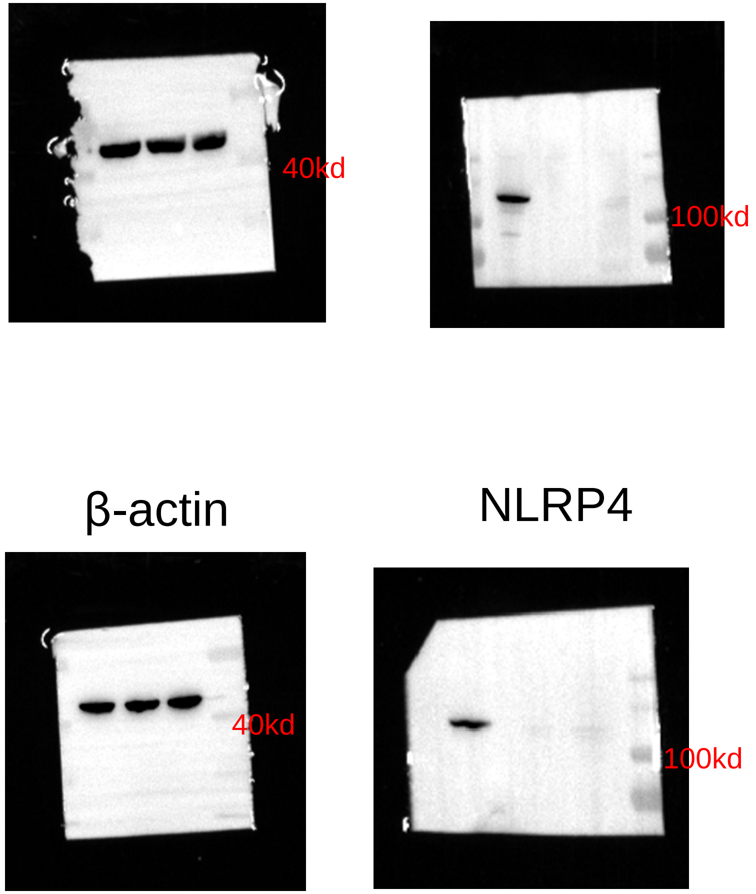

Fig 2j

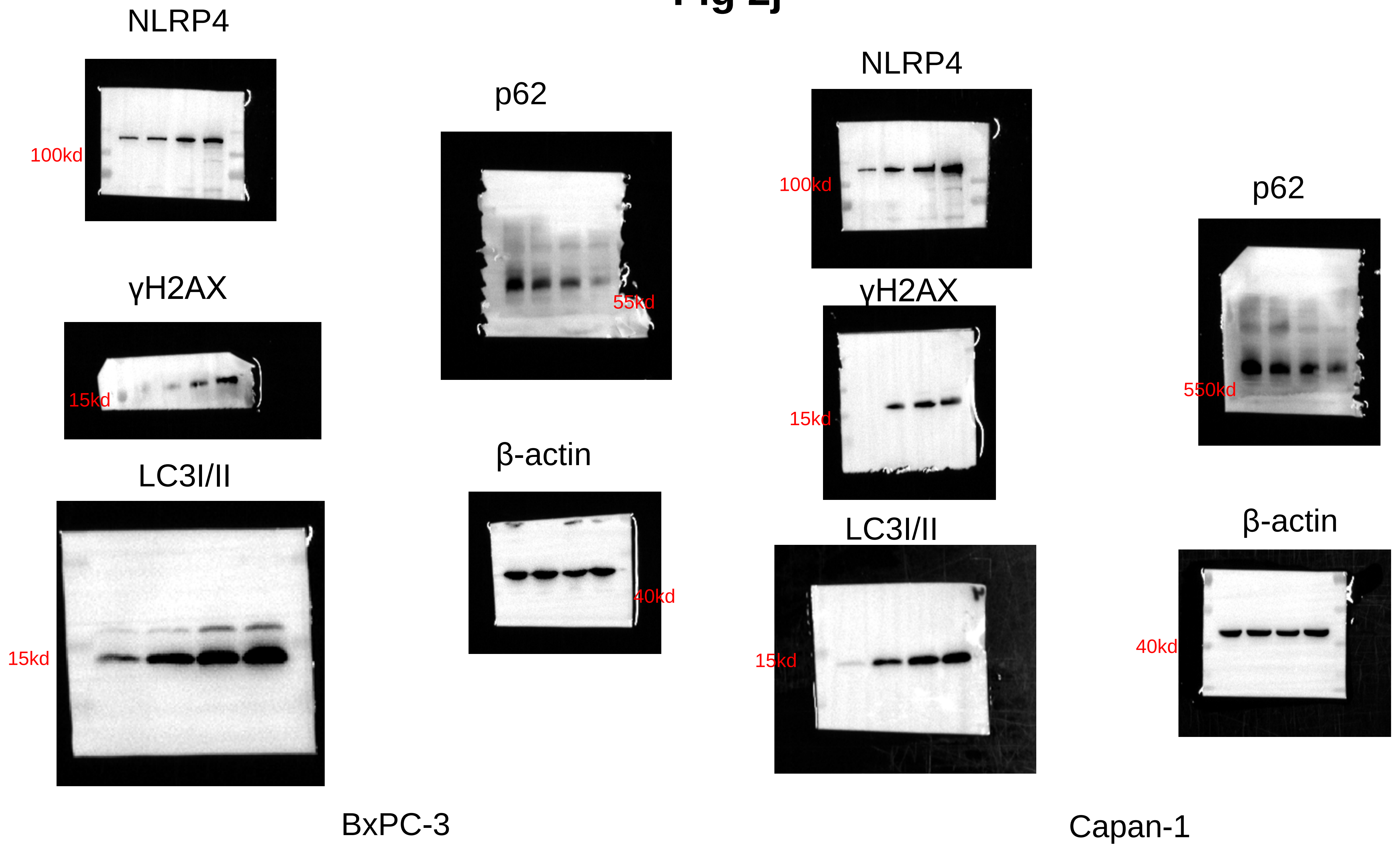
NLRP4
100kd
p62
55kd
γH2AX
15kd
LC3I/II
15kd
β-actin
40kd
BxPC-3
NLRP4
100kd
γH2AX
15kd
LC3I/II
15kd
p62
550kd
β-actin
40kd
Capan-1

Fig 3d

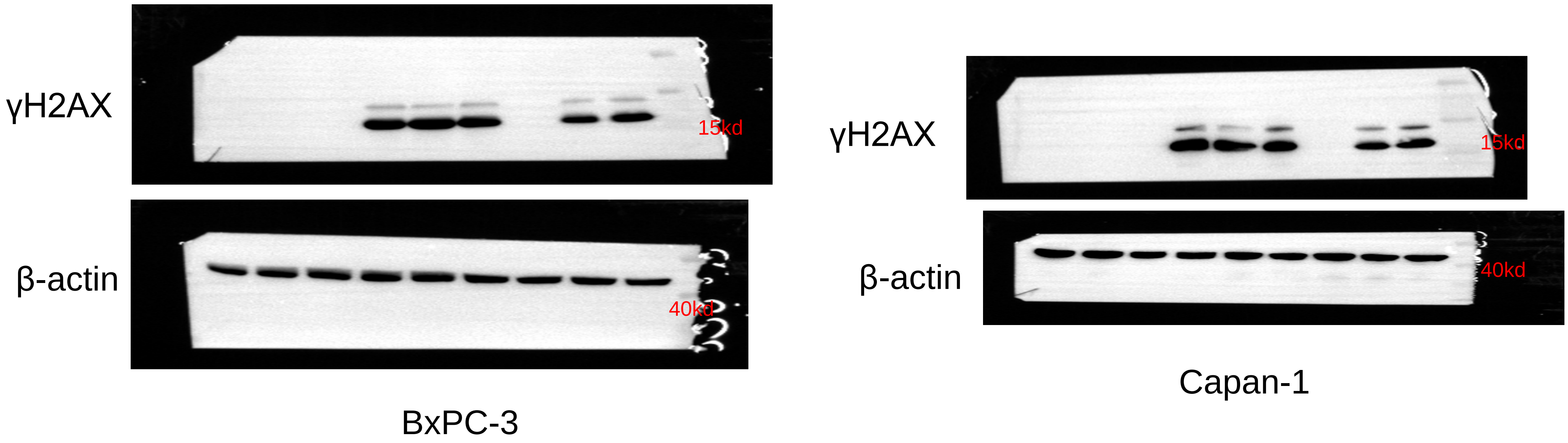

Fig 4a

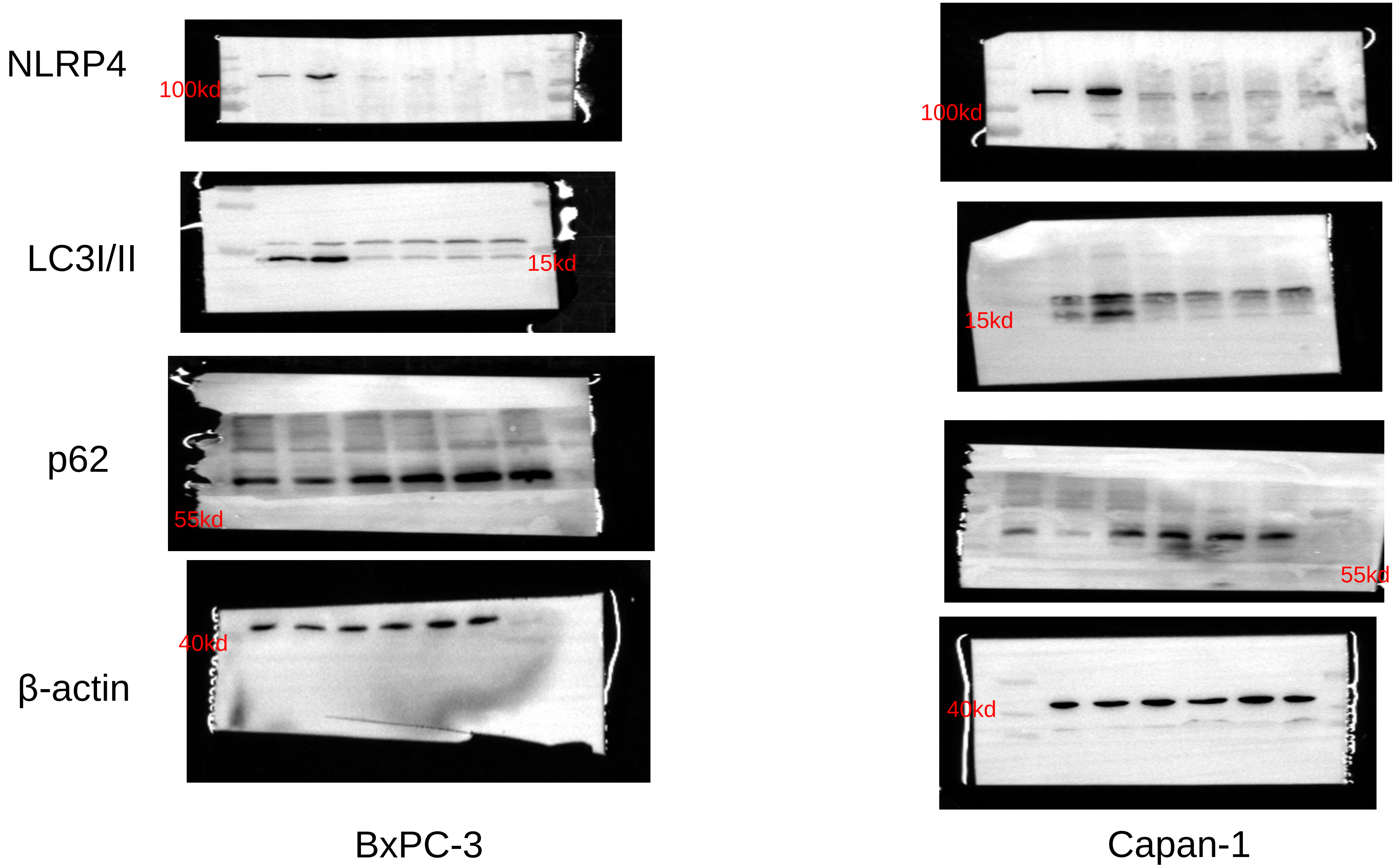
NLRP4
LC3I/II
p62
β-actin
100kd
15kd
55kd
40kd
100kd
15kd
55kd
40kd
BxPC-3
Capan-1

Fig 5j

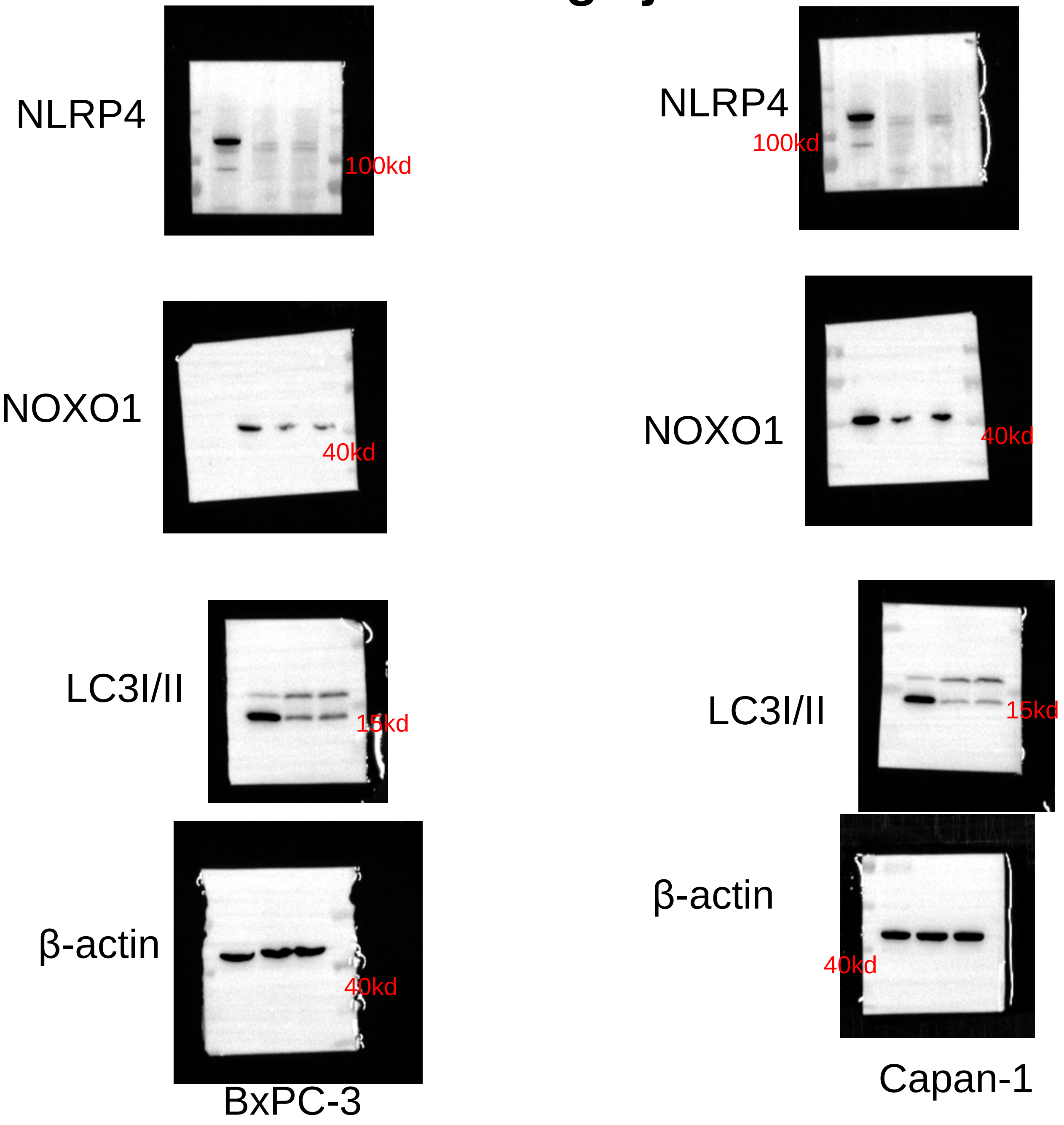
NLRP4
100kd
NOXO1
40kd
LC3I/II
15kd
β-actin
40kd
BxPC-3
NLRP4
100kd
NOXO1
40kd
LC3I/II
15kd
β-actin
40kd
Capan-1

Fig 6c

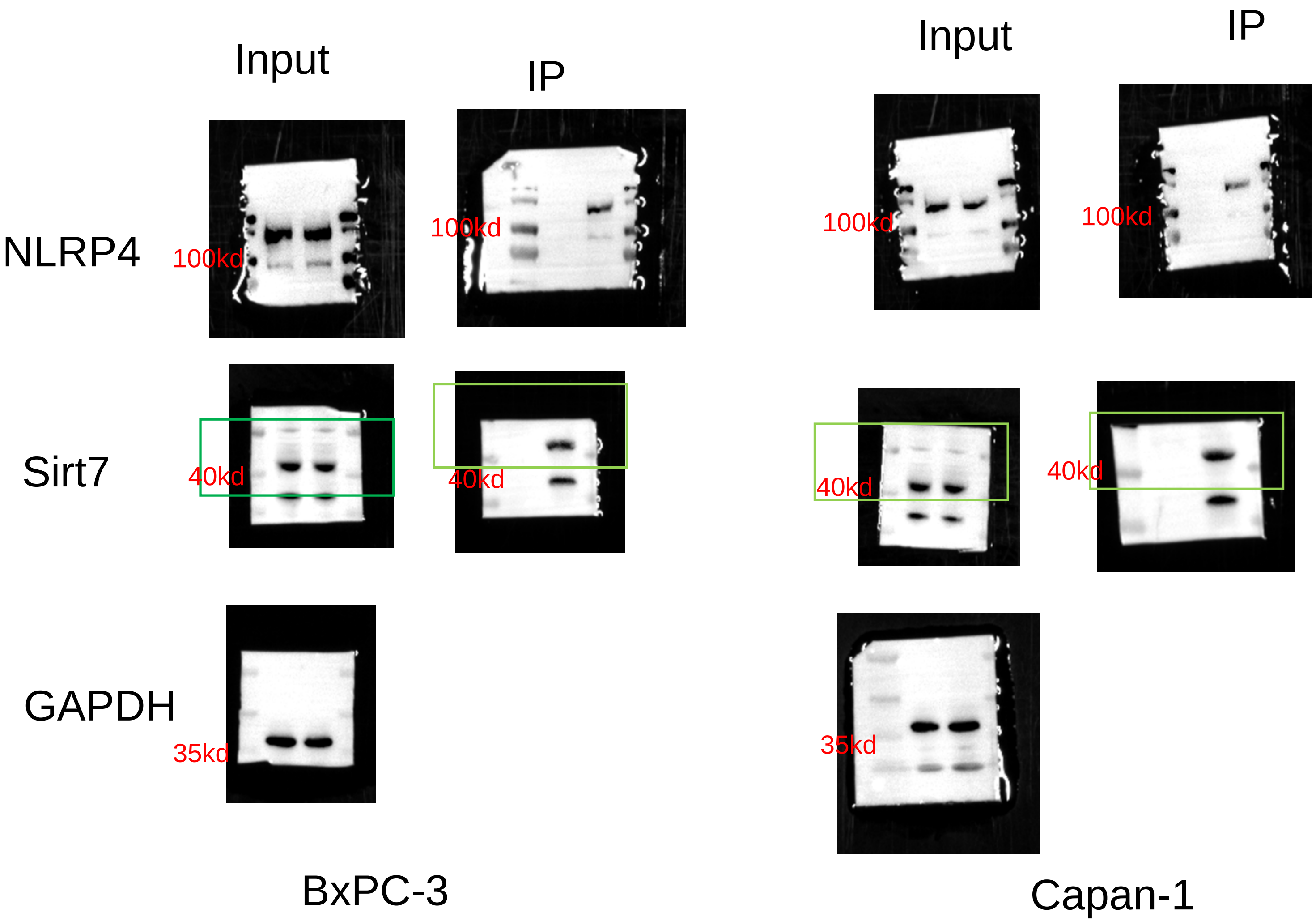
Input
IP
Input
IP
NLRP4
100kd
100kd
100kd
100kd
Sirt7
40kd
40kd
40kd
40kd
GAPDH
35kd
35kd
BxPC-3
Capan-1

Fig 6d

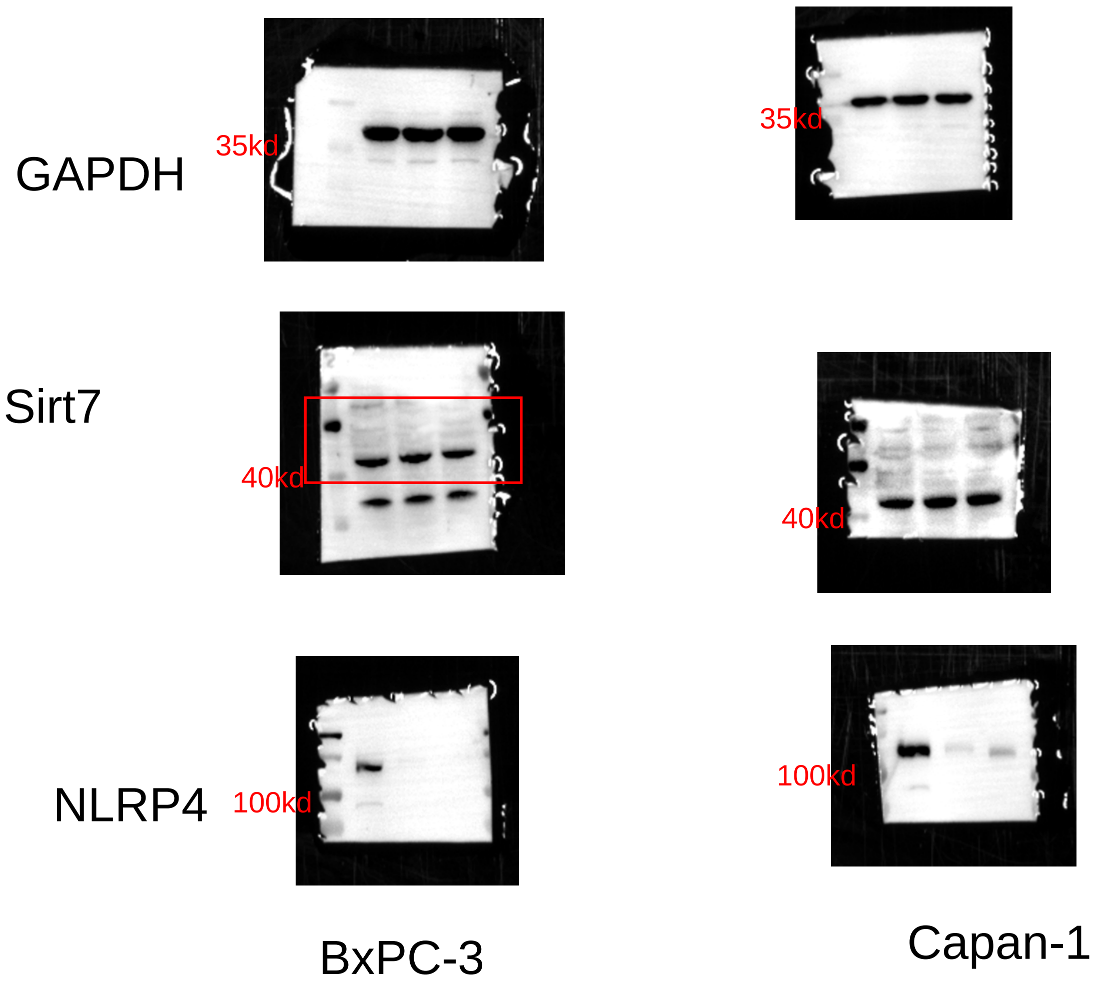
GAPDH
35kd
35kd
Sirt7
40kd
40kd
NLRP4
100kd
100kd
BxPC-3
Capan-1

Fig 6e

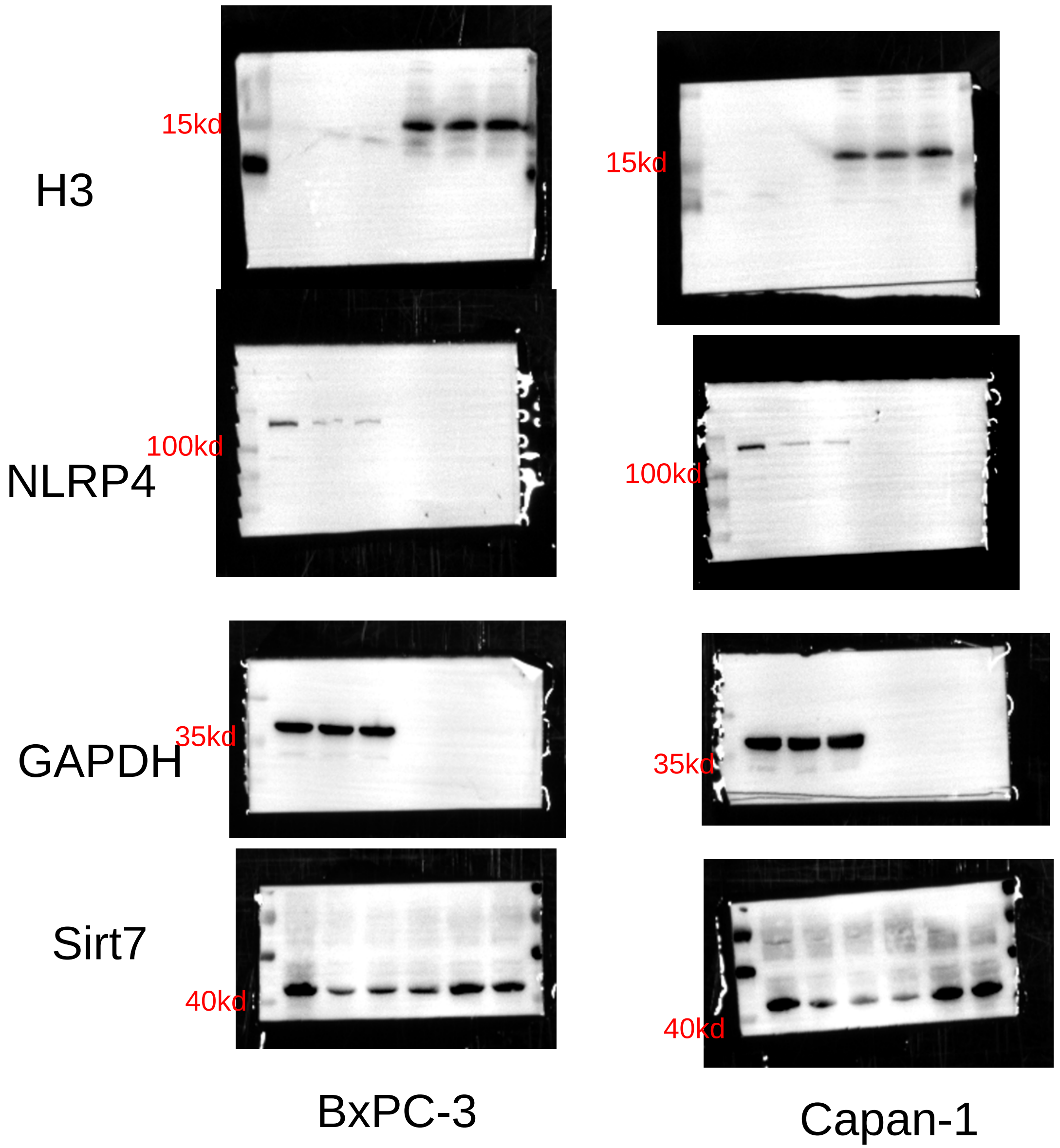

H3
15kd
15kd
NLRP4
100kd
100kd
GAPDH
35kd
35kd
Sirt7
40kd
40kd
BxPC-3
Capan-1

Fig 8j

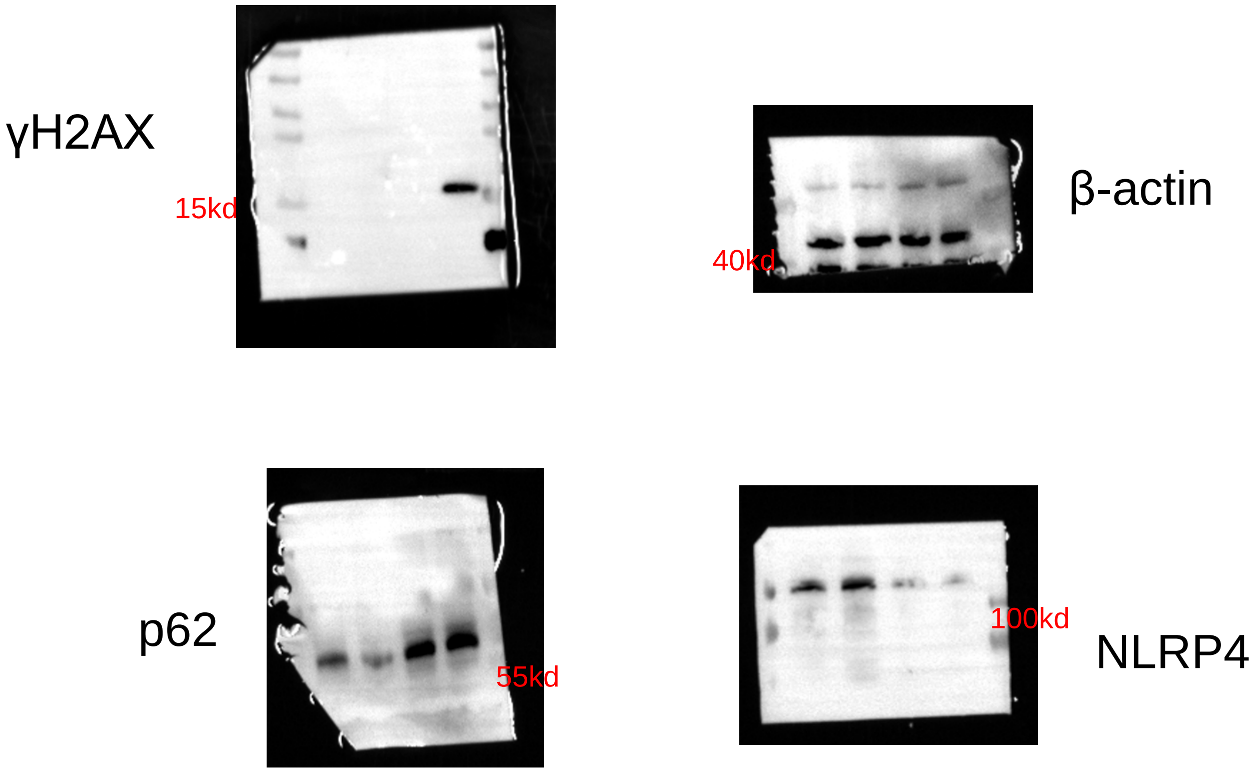

Fig 9c

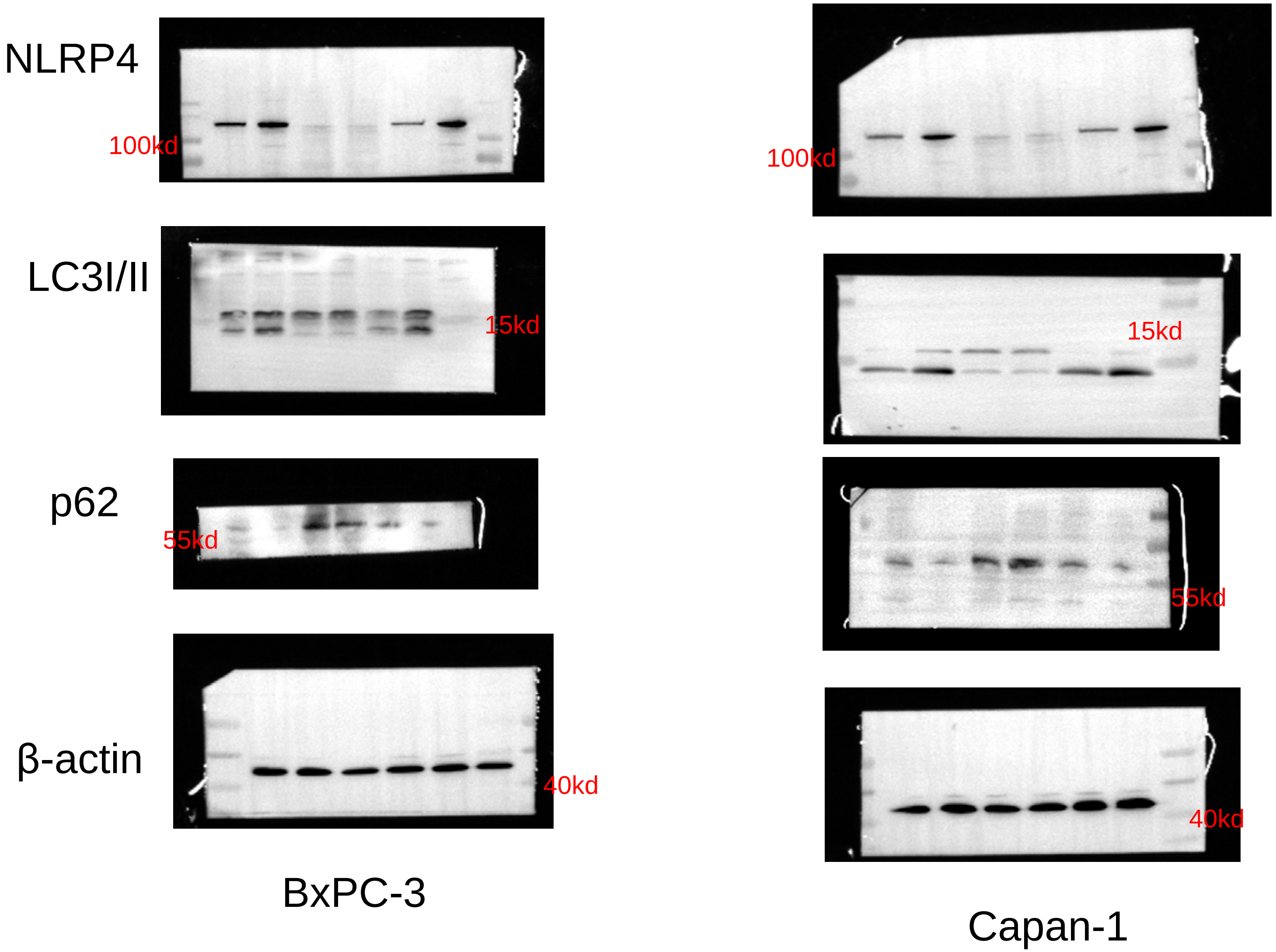

NLRP4
LC3I/II
p62
β-actin
100kd
15kd
55kd
40kd
BxPC-3
100kd
15kd
55kd
40kd
Capan-1

Extended Data Fig. 9a

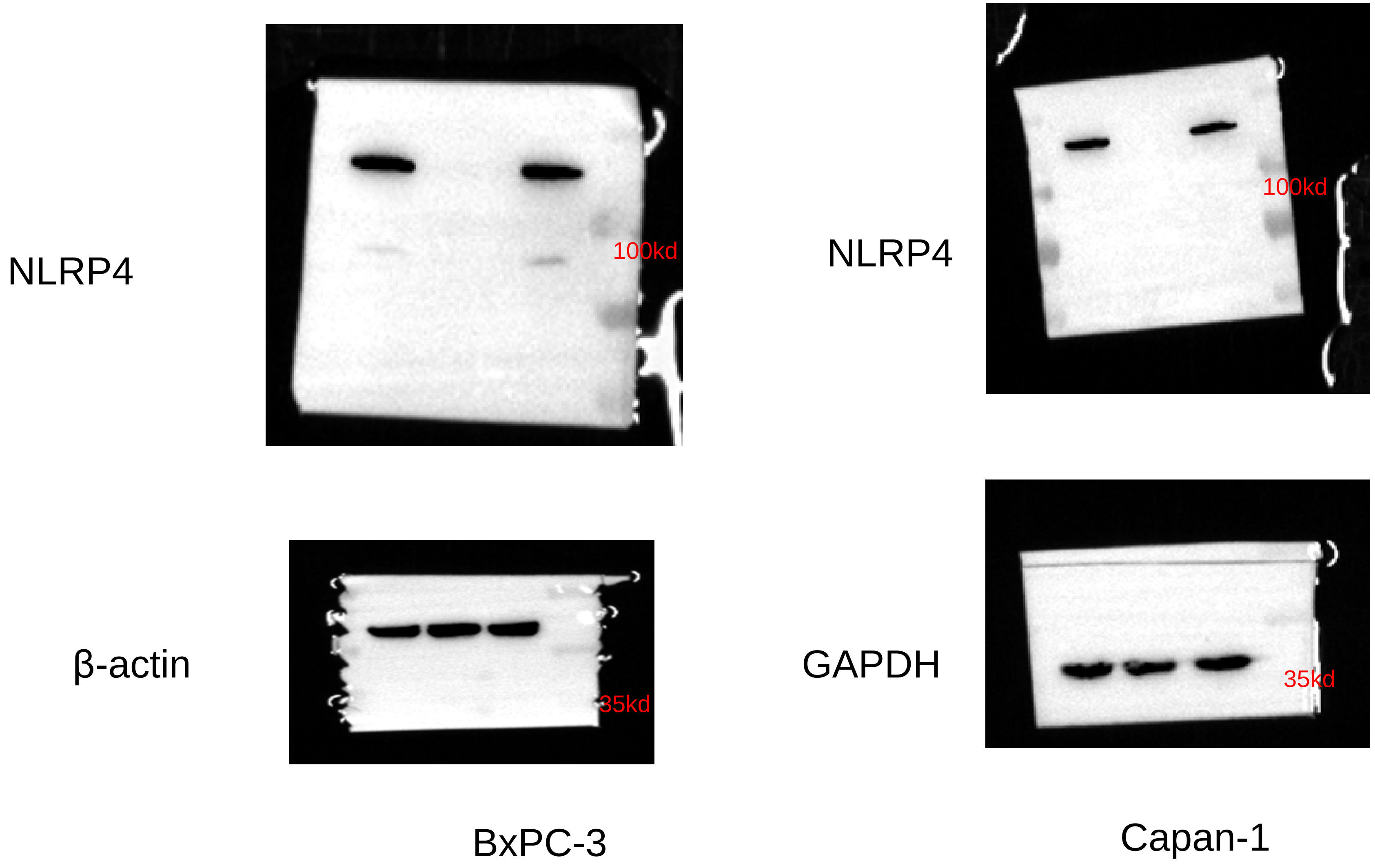

Supplement: Supplementary file 2 — Original data files [file 41419_2024_6984_MOESM2_ESM.pdf]
